# Numerical Investigations on Seismic Behavior of Segmental Assembly of Concrete Filled Steel Tube Piers with External Replaceable Energy-Dissipating Links

**DOI:** 10.3390/ma16031122

**Published:** 2023-01-28

**Authors:** Chengquan Wang, Chongli Yin, Yun Zou, Boyan Ping, Xi Wu, Juan Liao, Miaomiao Sun

**Affiliations:** 1 Department of Civil Engineering, Hangzhou City University, Hangzhou 310015, China; 2 Zhejiang Engineering Research Center of Intelligent Urban Infrastructure, Hangzhou 310015, China; 3 Key Laboratory of Safe Construction and Intelligent Maintenance for Urban Shield Tunnels of Zhejiang Province, Hangzhou 310015, China; 4 School of Environment and Civil Engineering, Jiangnan University, Wuxi 214122, China; 5 Department of Civil Engineering, Tongji University, Shanghai 200092, China

**Keywords:** segmental assembly pier, concrete filled steel tube pier, external replaceable energy-dissipating link, seismic performance, self-centering, energy dissipation

## Abstract

In order to reduce the damage sustained by the substructure of bridges during an earthquake, reduce economic loss, avoid casualties, and ensure the quick repair of bridges after an earthquake, this paper, inspired by the good seismic performance of the rhombic opening in the shear wall structure, proposes a precast segmental concrete-filled steel tubular (PSCFST) pier with external replaceable energy-dissipating links (EREDL).Through finite element simulation analysis, it can be found that the energy dissipation capacity of a PSCFST pier with external EREDL is increased by 104% compared with that of a PSCFST pier without EREDL, and the lateral bearing capacity is increased by 76.9%. Through parameter analysis, it can be found that the change of initial prestress has little effect on the energy dissipation capacity of PSCFST piers, and the seismic performance of PSCFST piers can be improved by properly increasing the ultimate tensile strength of the energy dissipator materials. Compared with the energy dissipators made of Q235 steel, the energy dissipation capacity of PSCFST piers made of Q435 steel energy dissipators is increased by about 85.4%; At the same time, the thicker the energy dissipator, the stronger the energy dissipation capacity of the PSCFST pier, and the lateral bearing capacity is further improved.

## 1. Introduction

In recent years, precast bridge piers have developed rapidly, which has attracted extensive attention. The main advantages of precast bridges include cleaner construction, reduced harm to the environment, and reduced construction time [1,2]. Compared with traditional piers, precast assembled piers can effectively reduce residual displacement after an earthquake due to an excellent self-centering ability [3,4]. However, precast assembled piers also have disadvantages; more specifically, the pier-shaping hinge area can be seriously damaged during an earthquake, and their energy consumption capacity is low, which makes them unsuitable for high-intensity areas [5].

In recent years, seismic engineering research has gradually shifted its attention from seismic isolation to the self-centering ability of buildings and structures [6]. In order to enhance the energy dissipation and self-centering capacity of buildings, many scholars have carried out a number of studies, and a variety of seismic structures have been designed and engineered. In 1997, Mander et al. [7] pioneered the concept of a self-centering pier structure. It was the first time that a rocking structure, precast assembly technology, and unbonded prestressed reinforcement connections had been applied to bridge piers. Pseudo-static test research has been conducted on segment precast assembled piers under different conditions. Test results showed that a precast pier with prestressed reinforcement has excellent seismic resilience and small structural damage, but the energy consumption capacity is insufficient. In 2002, Hewes et al. [8] conducted a test to investigate a precast single pier with steel sleeves at the bottom segment under a simulated horizontal seismic action. It was found that, compared with the stirrup densification of traditional piers, the steel tube had a better restraining effect on the concrete in the plastic hinge area. However, the plastic length increased and caused more serious damage to the upper segment. In 2006, Hewes, Chou, and Chen [9] conducted a comparative study of quasi-static tests on two groups of pier specimens with energy dissipation devices. The results showed that the lateral restraint capacity and energy dissipation capacity of the segment are enhanced by the application of the energy dissipation device. In 2016, Varela et al. [10] set up rubber seismic isolation bearings at the joints between the pier body and the bearing platform, and the precast pier and the bearing platform were combined into a whole with shape memory alloy. It was found that setting the energy dissipation device at the key energy-consuming position greatly improved the energy consumption capacity of the prefabricated pier. In 2017, Mehrshad et al. [11] proposed a new energy-dissipating device composed of a lead plate and a steel plate, which are installed at the joint between the bottom and the pile of segmental assembly pier, and the pier bottom-shaping hinge segment is constrained by a steel tube and subjected to a quasi-static test. The results showed that the energy dissipation capacity of the pier was significantly improved, and the concrete damage at the bottom of the pier was not obvious due to the constraint of the steel tube. In 2020, Qi Zhang et al. [12] divided the seismic-resistant precast bridge columns into three categories: emulative column, simple rocking column, and hybrid rocking column. They pointed out that the external energy dissipation rod of the hybrid rocking column can improve the energy dissipation capacity of the column. Compared with the other two types, the hybrid rocking column is undoubtedly the better choice. In addition, Qi Zhang et al. gave a recommended design effective stiffness ratio through existing tests and proposed a regression equation for predicting residual drift vs. maximum drift ratio and viscous damping ratio. In 2022, Xueqi Zhong et al. [13] proposed a rocking mechanical hinge (RMH), which uses post-tensioned tendons to provide recentering capability, and oval steel dissipaters to dissipate energy. The seismic performance of a RMH column, conventional reinforced concrete (RC) column, and conventional rocking column was studied using a nonlinear time history analysis. The results showed that the RMH column had superior seismic performance. Compared with a traditional RC column, the residual displacement can be disregarded, and there is less local damage when compared with a traditional rocking column. From 2019 to 2022, Yuan Wancheng et al. [14] and Zhang et al. [15] poured the bottom of the pier and the cap to form a whole, assembled the segments on the upper part of the pier, and applied prestress to form a mixed system. The study found that using cast-in-place for the bottom section of the pier and for the pile caps to form a whole can avoid the adverse effects of pier bottom joints on the development of plastic dumplings. Applying prestress to the equivalent cast-in-place system to form a hybrid system can effectively improve the self-centering ability of assembled bridge piers and significantly improve seismic performance.

In order to enhance the energy dissipation and self-centering capacity of building structures, researchers have proposed a variety of energy dissipation devices and structural transformation, and a number of studies have been carried out. Metallic dampers are widely used as energy dissipation devices in buildings because of their low manufacturing cost, stable hysteresis characteristics, environmental temperature resistance, high reliability, and strong energy dissipation capacity [16]. Since then, diamond-shaped holes have been widely used in various energy dissipation structures due to their energy dissipation capacity, clear damping mechanism, and stable performance. In 2012, Wang Shuang [17] used ABAQUS to simulate the low-cycle reciprocating quasi-static loading of five types of perforated H-steel dampers designed with different holes. The analysis results showed that the diamond-shaped perforated H-steel damper has the largest equivalent damping ratio and the most uniform stress distribution. In 2018, Lin Yuhui [18] conducted a nonlinear reciprocating loading quasi-static test on a steel plate wall with rhombic openings. The results showed that when the width to height ratio of the opening increased, the initial stiffness and bearing capacity of the steel plate wall increased, but the degradation rate of the bearing capacity and stiffness accelerated, and the energy dissipation capacity weakened. In 2019, Xu Li and Jiang Chao innovatively proposed an improved “rhombic multi-hole steel plate damper” as a replaceable coupling beam damper. Using ANSYS to analyze the damper, it was found that, compared with slot type and double X-shaped mild steel dampers, the rhombic hole damper had the largest shear stiffness, and the major axis of the rhombic hole and the thickness of the steel plate of the damper had the largest impact on the damping force of the damper; however, the thickness of the steel plate had little effect on the maximum stress of the damper [19]. In 2022, Wang et al. [20] designed a rhombic opening connection device for the pier joint and used ABAQUS finite element software to conduct a quasi-static analysis of the device. The results showed that improving the section ratio can improve the lateral bearing capacity and energy dissipation capacity of the pier. When the section ratio increased to 4%, the energy dissipation capacity of the CFST pier increased by 77.8%, and the lateral bearing capacity increased by 33.9%.

Yasin Onuralp Özkılıç et al. [21] presented an experimental and numerical study undertaken to investigate the performance of extended unstiffened and stiffened end-plate connections used in replaceable shear links. The results demonstrated that end-plates designed according to the AISC guidelines or Eurocode provisions demonstrated acceptable performance in terms of the target link rotation angle. Due to strain-hardening effects, thinner plates than the ones suggested by the codes were also found to demonstrate satisfactory performance. Yasin Mehmet Bakir Bozkurt et al. [22] presented a novel, detachable, replaceable link which employs a splice connection at the mid-length of the link. Proof-of-concept testing of the proposed links was performed on 3 specimens where the type of force transfer in the splice connection was considered as the prime variable. All specimens failed at link rotation angles that were significantly higher than the link rotation angle required by AISC341 and demonstrated the potential of the proposed link concept. Complementary finite element parametric studies were conducted to validate the design procedure developed for the proposed replaceable link concept. Ozkilic et al. [23] examined different stiffener configurations. The main objective was to improve the behavior of short links using different stiffener configurations. Pursuant to this goal, a comprehensive numerical study was conducted using ABAQUS. Based on the results, the stiffener configuration with two vertical and two diagonal stiffeners perpendicular to each other was recommended. The proposed stiffener configuration can increase the shear capacity, energy dissipation capacity, and the ratio of energy/weight up to 27%, 38% and 30%, respectively. Detailing of the proposed stiffener configuration was presented.

It can be found through the above research that, at present, the research on concrete-filled steel tube structures is relatively mature. However, when a concrete-filled steel tube is used in PS bridge piers, its mechanical performance, seismic mechanism, and theoretical calculation methods still need more detailed research, especially for PSCFST bridge piers that give consideration to damage control, self-centering, and repairability. In this paper, the concept of recoverability is introduced into the seismic design of a PS pier, and a PSCFST pier with recoverable function is proposed and designed. Through research, the seismic mechanism, damage development mechanism, and segment interface stress mechanism of a PSCFST pier are revealed, and the seismic design method of a PSCFST pier based on recoverable function is mastered to achieve the research goal of giving consideration to damage control, self-centering, and repairability, as well as to achieve the pier construction efficiency and the simultaneous improvement of seismic performance and post-earthquake resilience. Additionally, this paper further analyzes the impact of different EREDL parameters on the seismic performance of PSCFST piers. By establishing the ABAQUS finite element model and simulating its low-cycle pseudo static loading, the feasibility of EREDL–PFCSFT piers is discussed by comparing the finite element results with the seismic performance of PSCFST piers without EREDL. In addition, this paper also analyzes the impact of the initial prestress of the pier, EREDL strength, and EREDL thickness on the seismic performance of EREDL–PSCFST pier, focusing on the nonlinear force displacement relationship, inter-segment openings, segment-residual displacement, stiffness degradation, etc. of an EREDL–PSCFET pier in the process of horizontal reciprocating loading, puts forward the calculation formula of bending bearing capacity, and verifies its accuracy by comparing it with the finite element simulation results.

## 2. Establishment of PSCFST–EREDL

### 2.1. Model Design

**Figure 1:**
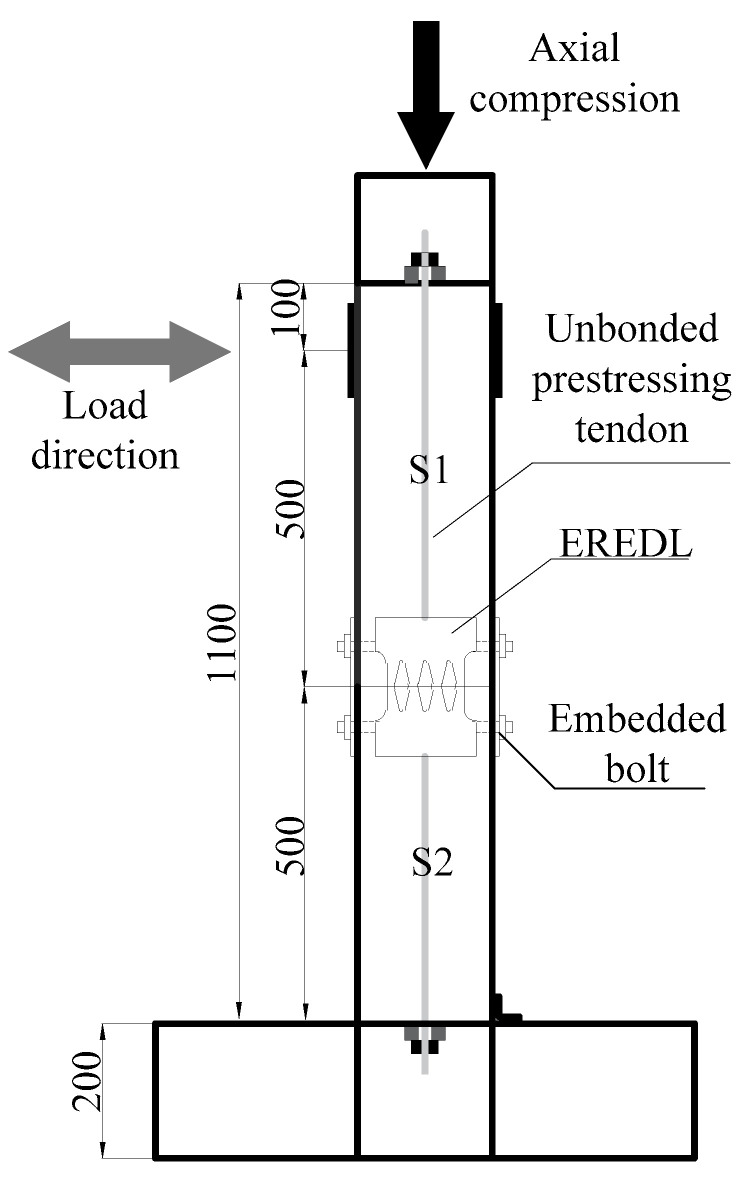
Construction of segmental assembled PSCFET bridge pier specimens.

**Figure 2:**
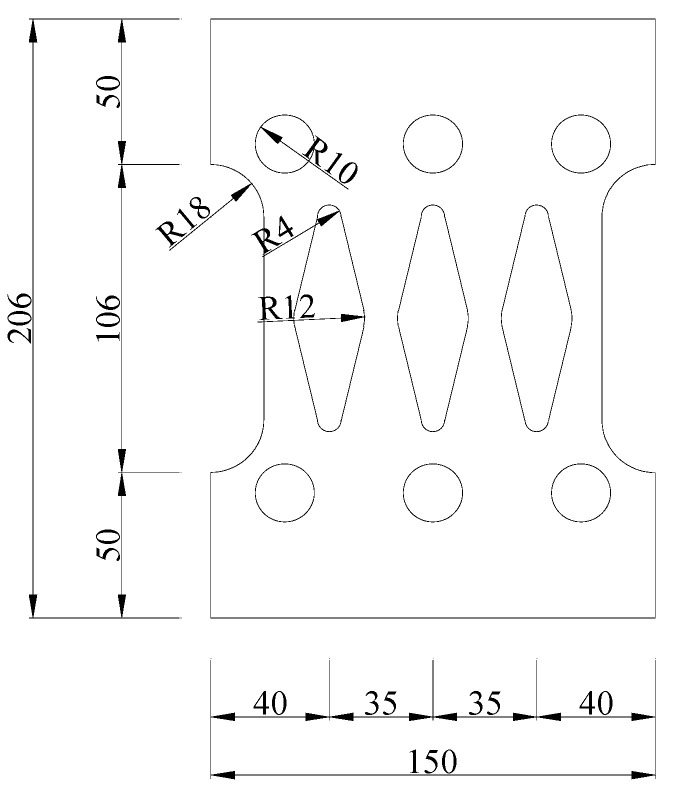
Diagram of EREDL (unit: mm).

In order to explore the feasibility of EREDL–PSCFST piers and the influence of initial prestress, EREDL strength and EREDL thickness on the seismic performance of piers, nine pier models were designed, each under different conditions. All the working conditions are shown in Table 1. The test piece is composed of a segmental steel tube, core concrete, unbonded prestressed reinforcement, EREDL, etc., as shown in Figure 1. The pier shaft of the EREDL–PSCFST pier is divided into two sections, S1 and S2. The size of the steel tube sections are 200 mm, 200 mm, 500 mm, respectively, and the wall thickness is 20 mm. Q345 steel is used for the steel tube section, and the concrete grade is C40. EREDL is set at the joint of the S1 and S2 segments, 60 mm from the upper and lower ends of the joint. The EREDL is made of Q235 steel with a thickness of 10 mm. A diamond shaped hole is set in the middle, which narrows gradually from both ends to the middle, to avoid stress concentration damage at the screw hole, and to ensure that the weakest position is in the middle of the energy consuming parts. The size of the structure is shown in Figure 2.

**Figure 3:**
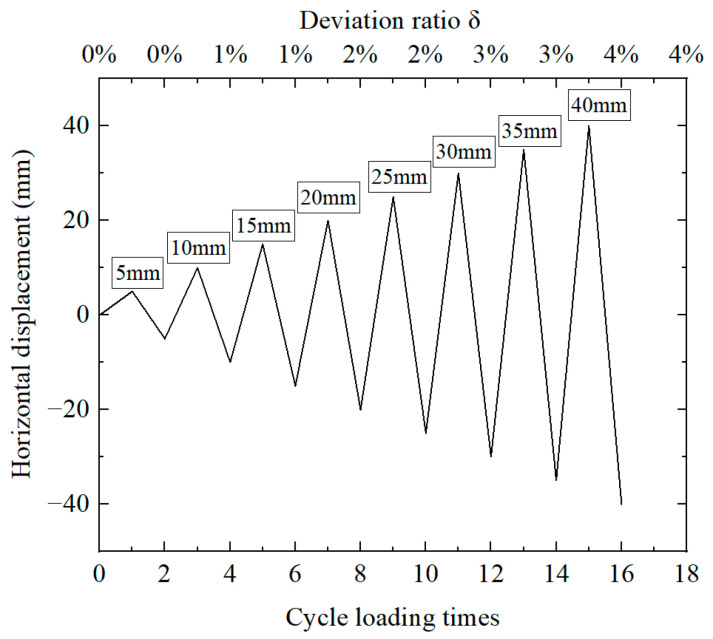
Displacement loading scheme.

The pier cap and cushion cap are connected by 715.2 unbonded prestressed steel strands, and the applied prestress is 600 MPa. In a finite element model, each component uses the corresponding material properties, and the loading method is low-cycle reciprocating loading. The loading position is in the center of the side of the fixture, and the bottom of the pier column is fully constrained to form a cantilever structure. The loading mode adopts displacement control, loading to the design deviation ratio of 4%, and the loading amplitude increases from 5 mm to 40 mm, in turn, with one loading cycle per stage. The loading curve is shown in Figure 3.

### 2.2. Constitutive Relationship

The constitutive relation of steel tube material adopts the mixed model of metal material kinematic hardening. The kinematic hardening model is used to simulate the behavior of independent equivalent pressure materials under cyclic loading, and can be used for general metals.

The finite element model in this study is a nonlinear isotropic tunnel reinforcement hybrid model, and the reinforcement part in the calculation formula includes two parts:

First, the nonlinear kinematic hardening component of yield surface movement is described by back stress. The equivalent yield surface is defined as:

(1) $$F=f\left(\sigma -\alpha \right)-\sigma^{0}$$

where $\sigma^{0}$ is the yield stress and the equivalent Mises potential function about the back stress α, as shown in Formula (2):

(2) $$f\left(\sigma -\alpha \right)=\sqrt{\frac{3}{2}\left(S-\alpha^{dev}\right):\left(S-\alpha^{dev}\right)}$$

where S=σ+pI is the deviatoric stress tensor, σ is the stress tensor, p is the equivalent compressive stress, I is the unit tensor, and $\alpha^{dev}$ is the deviator stress tensor.

The other is the nonlinear isotropic hardening component used to describe the size of the yield surface. The kinematic hardening component is an additional relaxation variable on the basis of the Ziegler strengthening criterion, and the influence of multiple back stress effects can also be considered. The form of the back stress increment is:

(3) $$d\alpha_{k}=C_{k}\frac{1}{\sigma^{0}}\left(\sigma -\alpha \right)d{\bar{\varepsilon }}^{pl}-\gamma_{k}\alpha_{k}{\bar{\varepsilon }}^{pl}$$

It can be written as:

(4) $$\alpha_{k}=\frac{C_{k}}{\gamma_{k}}\left(1-e^{-\gamma k{\bar{\varepsilon }}^{pl}}\right)$$

where total back stress $\alpha =\sum_{k=1}^{N}\alpha_{k}$, N is the amount of back stress. $C_{k}$ and $\gamma_{k}$ material parameters need to be calibrated through tests. $C_{k}$ is the initial strengthening quantity, and $\gamma_{k}$ determines the decrease in the dynamic hardening modulus with the increase in plastic strain. The kinematic hardening criterion can be divided into two parts: the inlet pressure shaft part and the off-plane part. Only the off-plane part is affected. When $C_{k}$ and $\gamma_{k}$ are equal to zero, the model degenerates into an isotropic hardening model. When $\gamma_{k}$ is zero, the model degenerates into a Ziegler reinforcement criterion.

The yield surface of Isotropic hardening $\sigma^{0}$ is defined as a function of equivalent plastic strain ${\bar{\varepsilon }}^{pl}$:

(5) $$\sigma^{0}=\sigma {|}_{0}+Q\left(1-e^{-b\cdot \bar{\varepsilon }pl}\right)$$

where $\sigma {|}_{0}$ is the yield stress at zero plastic strain, Q and b are material parameters, Q is the maximum change of yield surface, and b determines the reduction in the strengthening quantity with the increase in plastic strain.

On the basis of a steel tube material property test, the nominal stress-strain relationship curve obtained from the test is converted into the real stress-strain relationship curve. Then the true stress-strain relationship curve is transformed into the true stress-strain relationship curve (data input format in ABAQUS), and the smooth curve is obtained by fitting. From the material property test, the parameters of the mixed strengthening model (one back stress) are calibrated and analyzed, and the following parameter calculation formula is obtained:

Initial yield strength $\sigma {|}_{0}=0.85f_{y}$.
Maximum bearing strength $f_{yu}=2.1f_{y}$.
Kinematic hardening parameters C=0.02E.
Kinematic hardening parameter (M is 0.5 temporarily) $\gamma =C/\left[(f_{yu}-\sigma^{0}\right)M]$.
Isotropic hardening parameters $Q=(f_{yu}-\sigma^{0})\left(1-M\right)$.
Isotropic hardening parameters b=0.5C/Q.

The constitutive relation of concrete materials can be found in the previous study [20].

The concrete is simulated by the Concrete Damage Plasticity (CDP) model, and the plastic parameters of concrete are shown in Table 2. Where ψ is the expansion angle, ϵ is the flow potential offset value, $f_{b0}/f_{c0}\;$is the ratio of biaxial ultimate compressive strength to uniaxial ultimate compressive strength, $K_{c}$ is the invariant stress ratio, and μ is the viscosity coefficient.

The research shows that when the concrete uses the plastic damage model of concrete provided by finite element ABAQUS, the improvement of its restrained strength can be achieved by determining the yield surface function, but the improvement of the plastic property of concrete cannot be accurately simulated directly by finite element ABAQUS. The constitutive input of confined concrete into ABAQUS software can make up for this shortage. The confined concrete constitutive model includes the increase in peak strain and the improvement in ductility of the descending section of concrete due to the confinement of steel pipe to concrete.

**Figure 4:**
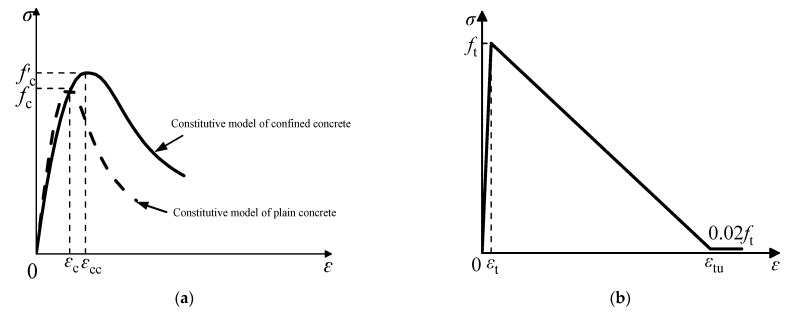
Stress-strain curves of concrete. (**a**) Compressive stress-strain relationship of concrete. (**b**) Tensile stress-strain relationship of concrete.

Based on this, the confined concrete compression model proposed by Han Linhai is selected as the concrete compression constitutive relationship in the CFST pier model, as shown in Figure 4a, and its expression is as follows:

(6) $$y=\left\{\begin{array}{l} 2x-x^{2}\;\left(x\leq 1\right)\\ \frac{x}{\beta_{0}{\left(x-1\right)}^{\eta }+x}\;(x>1) \end{array}\right.$$

(7) $$x=\frac{\varepsilon }{\varepsilon_{0}}$$

(8) $$y=\frac{\sigma }{\sigma_{0}}$$

(9) $$\sigma_{0}=f_{c}^{\prime }$$

(10) $$\varepsilon_{0}=\varepsilon_{c}+800\xi^{0.2}\times 10^{-6}$$

(11) $$\varepsilon_{c}=\left(1300+12.5f_{c}^{\prime }\right)\times 10^{-6}$$

(12) η=1.6+1.5/x

(13) $$\beta_{0}=\frac{{\left(f_{c}^{\prime }\right)}^{0.1}}{1.2\sqrt{1+\xi }}$$

(14) $$f_{c}^{\prime }=\left[0.76+0.2\log_{10}\left(\frac{f_{cu}}{19.6}\right)\right]f_{cu}$$

(15) $$E_{c}=4730\sqrt{f_{c}^{\prime }}$$

where$\;\beta_{0}$ is adjustment parameters of the descending section of the stress-strain curve of the concrete under compression, η is curve shape coefficient, $\varepsilon_{0},\;\varepsilon$ is peak strain of confined concrete and concrete strain, $\sigma_{0},\sigma$ is constraint concrete stress and concrete stress, $\varepsilon_{c}$ is peak strain of plain concrete, ξ is hoop coefficient, $f_{c}^{\prime }$ is compressive strength of concrete cylinder, and$\;E_{c}\;$is elastic modulus of concrete.

The tensile constitutive relationship of concrete is shown in Figure 4b, and its expression is as follows:

(16) $$f_{t}=0.26f_{cu}^{2/3}$$

(17) $$\varepsilon_{t}=65\times 10^{-6}f_{t}^{0.54}$$

(18) $$\varepsilon_{tu}=25\varepsilon_{t}$$

where $f_{t}$ is tensile strength of concrete, $f_{cu}$ is concrete cube compressive strength, $\varepsilon_{t}$ is strain corresponding to tensile strength of concrete, and $\varepsilon_{tu}$ is ultimate tensile strain of concrete.

### 2.3. Establishment of Finite Element Model

**Figure 5:**
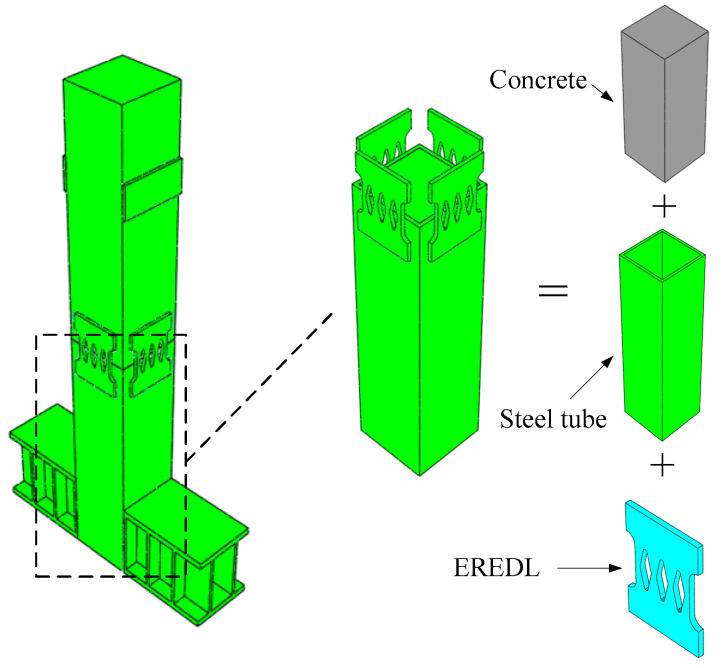
Finite element model of precast assembled pier.

In this paper, ABAQUS finite element software is used to establish the numerical model. Reduced integral element (C3D8R) is used for the pier body (concrete segment, steel tube, EREDL, and ground beam), and truss element (T3D2) is used for the simulation of unbonded prestressed reinforcements [24]. After a grid sensitivity analysis and comprehensive consideration of calculation efficiency and accuracy, the overall grid density of steel tube, concrete, and ground beam is 20 mm, the key part (the connection between EREDL and steel tube) is 10 mm, and the grid density of the EREDL is 1 mm. The contact between steel tubes and concrete, and between segments is surface–surface. The normal behavior is defined as “hard contact” that only transfers the pressure when the gap between the contact surfaces is 0; that is, the normal pressure is transferred when the surfaces are in contact, and the restraint of the nodes is invalid when the surfaces are separated. Tangential behavior between segments adopts a “penalty friction” which defines interface friction characteristics with a friction coefficient, and the friction coefficient is 0.4 [24]. Tie contact is used between the steel tube and the concrete, between the steel tube and the EREDL, and between the ground beam and the steel tube, as shown in Figure 5.

**Figure 6:**
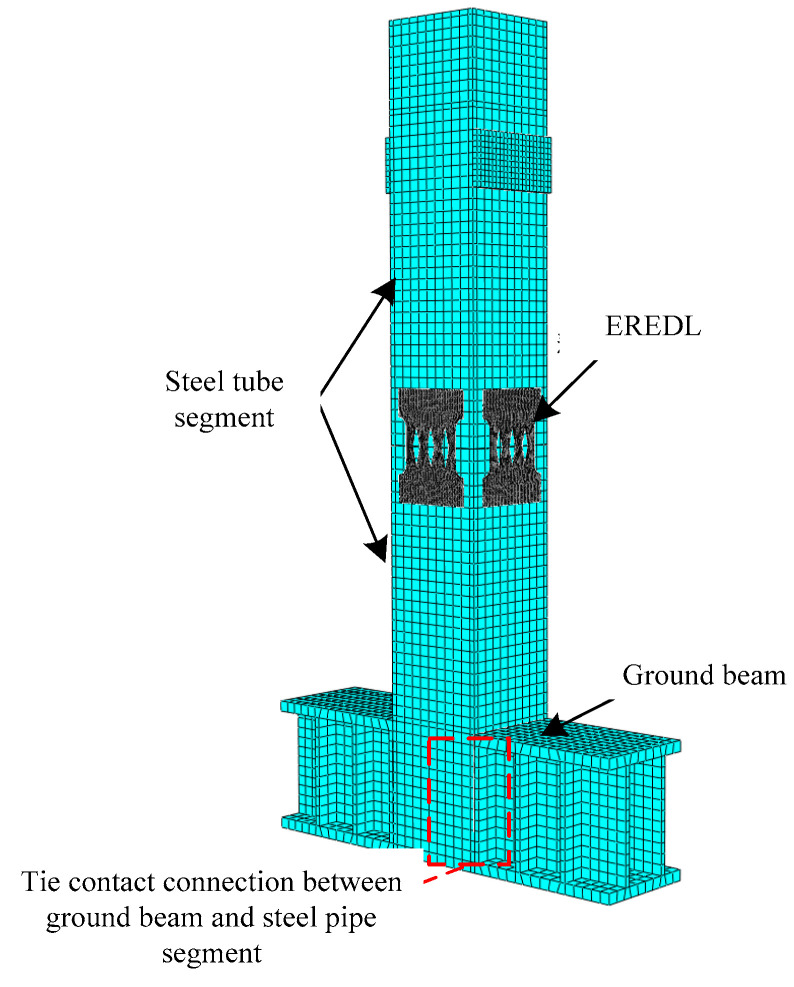
Finite element model of PSCFET.

The prestressed reinforcement is designed as an unbonded prestressed reinforcement, therefore the prestressed reinforcement is divided into three parts. The embedded connection is made between the prestressed reinforcement extending into the loading end and the foundation, and the other parts are not treated to simulate the unbonded state of the prestressed reinforcement between the concrete. Three steps are set in the analysis stage: the first step is to load the prestress, and the initial prestress of the basic model is set as 600 MPa. The prestress is applied through the cooling method, and the initial temperature and cooling value of the prestressed reinforcement are set, so that the initial prestress is set as the design value. The second step is to apply constant load axial pressure to all members, and the axial compression ratio is 0.15. In addition, a gasket is applied at the top of the column, which is defined as a rigid body to avoid local crushing of concrete. The third step is to carry out a lateral low cycle reciprocating loading. The loading scheme is displacement control, with an increment of 5 mm for each stage and one cycle for each stage. To facilitate the analysis, the piers in this paper are all set as cantilever structures, as shown in Figure 6; that is, all degrees of freedom are constrained at the bottom of the pier (Ux = Uy = Uz = 0), and there is no constraint at the top of the pier (Ux = Uy = Uz = 0). In order to obtain the reaction force at the bottom of the pier, a reference point is established at the center of the foundation base. A coupling method is used to couple the center of the foundation base and the reference point and constrain the translation and rotation degrees of freedom of the reference point in three directions to achieve the consolidation of the pier bottom. The established finite element analysis model is shown in Figure 6.

## 3. Model Validation

### 3.1. Test Introduction

**Figure 7:**
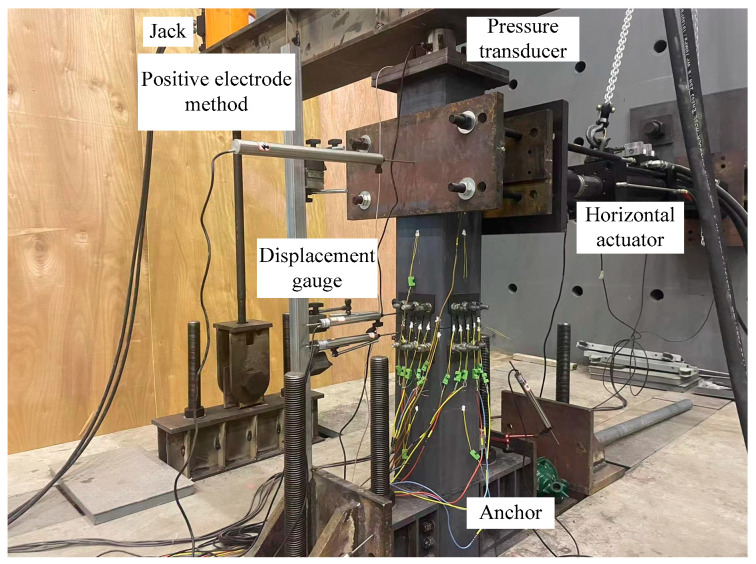
Measuring point arrangement.

**Figure 8:**
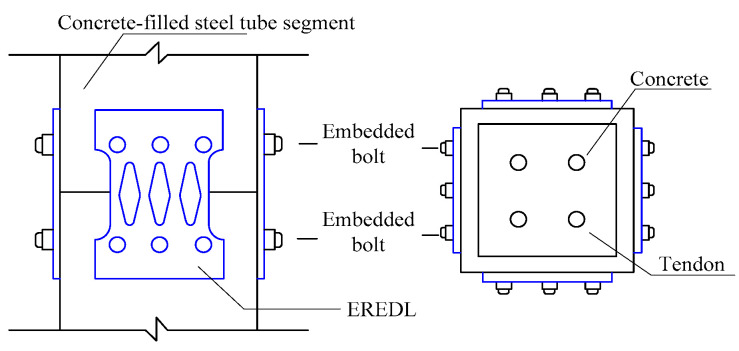
Connection structure diagram of EREDL.

In order to verify the feasibility and effectiveness of using the finite element method to establish the model for analysis, this paper conducted a reciprocating loading test on an EREDL-PSCFST pier [25]. The loading mode and mode are consistent with the finite element method. The layout of the loading device and strain gauge is shown in Figure 7. Where the EREDL is set at the joint of S1 and S2 segments, it is connected with S1 and S2 segments through embedded screws, which are not only used as an energy consuming device, but also improve the shear resistance of PSCFST piers. The connection structure of energy-consuming structures is shown in Figure 8. The specification of an unbonded prestressed steel strand connecting a pier cap and a cushion cap is 715.2. In the test, the concrete strength grade of the test piece is C40, and the measured compressive strength and tensile strength of the concrete cube are 42.5 MPa and 2.39 MPa, respectively (Table 3). The strength grade of the steel tube is Q345, the strength grade of the EREDL is Q235, and the thickness is 5 mm [26].

### 3.2. Comparative Verification

**Figure 9:**
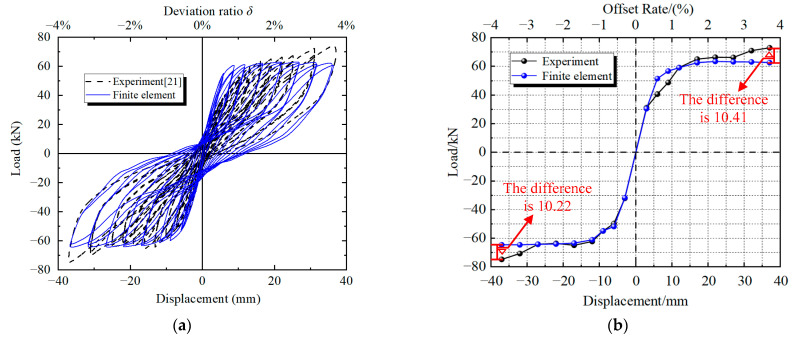
Comparison of hysteresis curves and skeleton curves [20]. (**a**) Hysteresis curve. (**b**) Skeleton curve.

Figure 9 shows the hysteretic curve obtained from the test and the numerical simulation of segmentally assembled PSCFET piers. When loading, the test results are in good agreement with the test results, and the specific comparison results are shown in Table 4. By comparing the horizontal bearing capacity, residual displacement, equivalent stiffness and energy dissipation, it can be seen that the numerical simulation results are similar with the test results, the errors are within a reasonable range, and the model has good reliability. The established finite element analysis model can be used to analyze the seismic performance of the segmentally assembled PSCFET piers with external energy dissipation devices.

**Figure 10:**
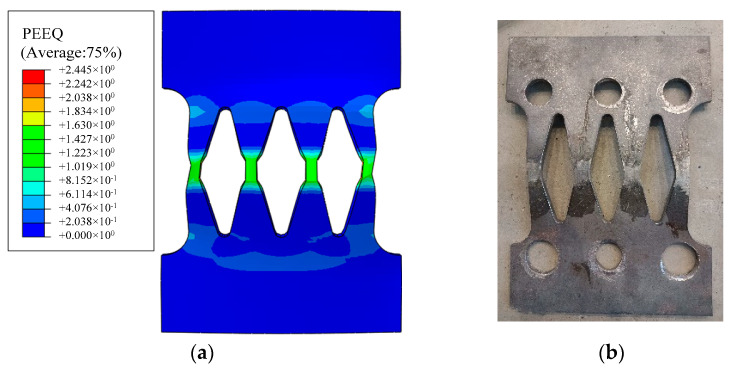
Comparison of EREDL deformation results. (**a**) Finite element model. (**b**) Experiment.

Figure 10 shows the comparison between the EREDL deformation diagram after the test loading and the EREDL deformation obtained from the finite element simulation analysis. It can be seen from Figure 10 that the deformation and damage laws of the finite element simulation results and the test results are basically consistent. Both have the middle opening part of the EREDL arched outward, and the deformation and damage are mainly concentrated in the diagonal part of the narrow rhombic hole. It can be seen that the finite element method can better simulate the deformation of the structure.

## 4. Finite Element Analysis

In order to explore the influence of the EREDL setting on the seismic performance of PSCFST piers, this section conducts research by comparing the hysteresis curve, energy dissipation capacity, cumulative energy dissipation, and stiffness degradation of two groups of PSCFST piers with or without EREDL, and explores its failure mechanism through the plastic damage nephogram of EREDL–PSCFST piers.

### 4.1. Hysteresis Curve

**Figure 11:**
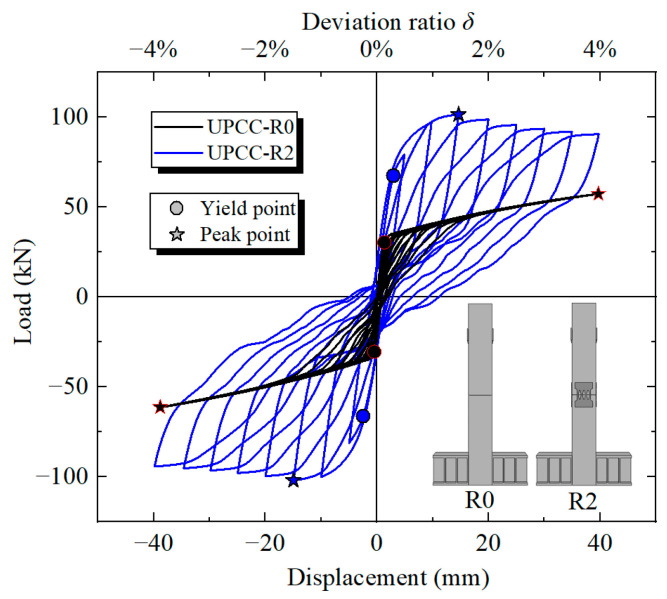
Comparison of hysteresis curves of two types of bridge piers.

Figure 11 shows the hysteresis curve and skeleton curve of a PSCFST pier without external EREDL and EREDL–PSCFST piers. Table 5 shows the skeleton curve characteristic values of the two piers. When the bearing capacity drops to 90%, the EREDL is basically completely yielded. Therefore, the horizontal loading displacement when the bearing capacity drops to 90% is the ultimate displacement of the structure, and the structure is destroyed. By comparing the hysteresis response of a PSCFST pier without an external EREDL with that of an EREDL–PSCFST pier, it can be found that: (a) The hysteresis curve of a PSCFST pier without an external EREDL is flag-shaped, the maximum bearing capacity is low (57.40 kN), the hysteresis loop area is small, and the energy dissipation capacity is poor; however, its residual displacement is small, indicating that the pier has good self-centering capacity. In the process of multiple loading, the change range of its reloading stiffness and unloading stiffness is small, indicating that its reloading stiffness and unloading stiffness are relatively stable, and the stiffness degradation is not obvious. (b) The hysteretic curve of an EREDL–PSCFST pier is relatively full, the area of the hysteretic loop is significantly increased, and the energy consumption capacity is significantly improved. The pier can accumulate more energy released by an earthquake. Affected by the sliding between segments, the EREDL has shear deformation, and the hysteretic curve has obvious pinch effect. Although the residual displacement is slightly increased, it remains small. The horizontal bearing capacity is set at the joint of the segment because the EREDL is set at the joint of the segment; therefore, the maximum bearing capacity of the bridge pier without an external EREDL is improved to 101.56 kN, which is about 76.9% higher.

By comparing the skeleton curve and its characteristic values, it can be seen that the yield strengths of the two specimens are 50.32 kN and 97.04 kN, respectively, and the R2 bearing capacity is higher than R0. The yield displacement of R2 is slightly less than that of R0 pier. It can be seen from Figure 11 that the initial stiffness and peak bearing capacity of R2 are higher than R0, mainly because the arrangement of the EREDL improves the lateral bearing capacity of the pier. Although the horizontal bearing capacity of the R2 specimen begins to degrade when the offset ratio is about 1.45%, the degradation speed is relatively slow, and when the offset ratio is 3.92%, the horizontal load of R2 still has 89.3% of the peak load. Japan Bridge (JRA 2012) stipulates [27] that the offset rate of bridge pier column residual displacement should not be greater than 1.0% to ensure that the column can be repaired. Although the residual displacement of the EREDL–PSCFST pier R2 test piece is larger than that of the PSCFST pier R0 test piece without the external EREDL, its positive residual displacement offset ratio is 1.09%, and the reverse residual displacement offset ratio is 0.48%, which can still ensure that the column can be quickly repaired after an earthquake.

### 4.2. Failure Mechanism

**Figure 12:**
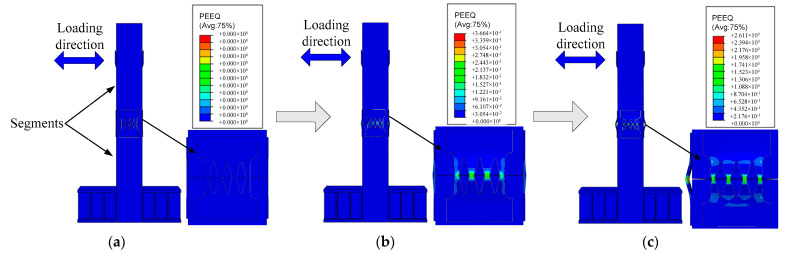
Deformation of specimen R0 during loading; (**a**) Prophase; (**b**) Metaphase; (**c**) δ=4%.

**Figure 13:**
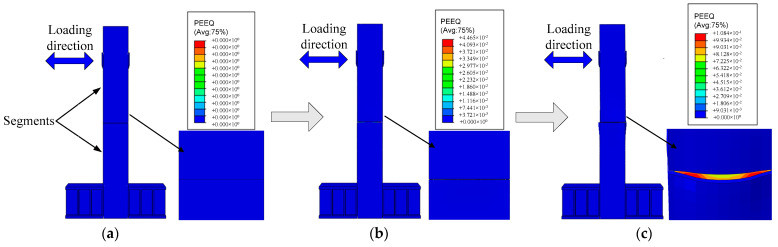
Deformation of specimen R2 during loading; (**a**) Prophase; (**b**) Metaphase; (**c**) δ=4%.

As shown in Figure 12 and Figure 13, when the deviation ratio of the previous specimen is small, the opening of the pier joint is small, and the setting of the connector has little effect on the deformation of the specimen. At this time, the R0 specimen has no obvious deformation damage, and there is a slight opening at the joint. There is no obvious deformation of piers and connectors in the R2 specimen. With the increase of the deviation ratio of the pier top, the joint of the R0 specimen opens obviously and increases continuously, and a small amount of concrete inside is crushed. The R2 specimen also has openings, but the opening degree is less than the R0 due to the setting of the connector; however, the weak area in the middle of the connector is deformed by tension. When the deviation ratio of the pier top of the specimen reaches 4%, the joint opening of the R0 specimen reaches the maximum, the dislocation occurs between the segments, the internal concrete is crushed, and the steel pipe at the joint is buckled. The joint opening of the R2 specimen is still less than the R0, and the steel tube has no obvious buckling deformation; however, the connector is basically yielding, and the deformation is obvious.

### 4.3. Energy Consumption Capacity

The energy dissipation coefficient and equivalent viscous damping coefficient are used to evaluate the energy dissipation performance of the bridge pier with or without an external EREDL. According to the calculation formula given in [27,28], the energy dissipation coefficient E and the equivalent viscous damping coefficient can be calculated by the area enclosed by the hysteresis curve:

(19) $$E=\frac{S_{\left(ABC+CDA\right)}}{S_{\left(OBE+ODF\right)}}$$

(20) $$h_{e}=\frac{1}{2}\times \frac{S_{\left(ABC+CDA\right)}}{S_{\left(OBE+ODF\right)}}$$

where is the area enclosed by the hysteresis curve, is the sum of the areas of and.

The energy consumption performance indexes of test pieces A and B are shown in Table 6. It can be seen from Table 6 that the energy dissipation coefficient of the pier without an external EREDL is 0.116, and the equivalent viscous damping coefficient is 0.018. The energy dissipation coefficient of the pier with an EREDL at the joint is 0.728, and the equivalent viscous damping coefficient is 0.116. The two indicators are significantly higher than those of the pier without an external EREDL, indicating that the setting of the EREDL can greatly improve the energy consumption capacity of the pier.

### 4.4. Cumulative Energy Consumption

**Figure 14:**
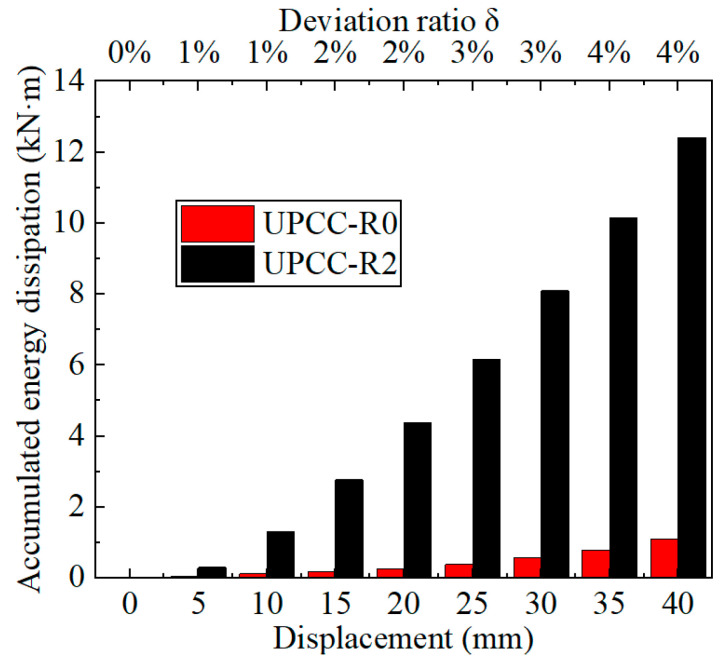
Cumulative energy dissipation curves of two types of piers.

Figure 14 shows the cumulative energy consumption curve of a PSCFST pier without an external EREDL and an EREDL–PSCSFT pier. Comparing the cumulative energy consumption curves of the two, it can be seen that the energy consumption capacity of the EREDL–PSCFST pier is significantly improved compared with that of the pier without an external EREDL. When the two test pieces reach the maximum displacement, the cumulative energy consumption of the pier without an external EREDL is 1.088 kN · mm, and the energy consumption capacity of an EREDL–PSCFST pier is 12.418 kN · mm; about 11.4 times of the former. The cumulative energy consumption of an EREDL–PSCFST pier in the later loading cycle is increasing because the EREDL can continue to play a role in the loading process.

### 4.5. Stiffness Degradation

**Figure 15:**
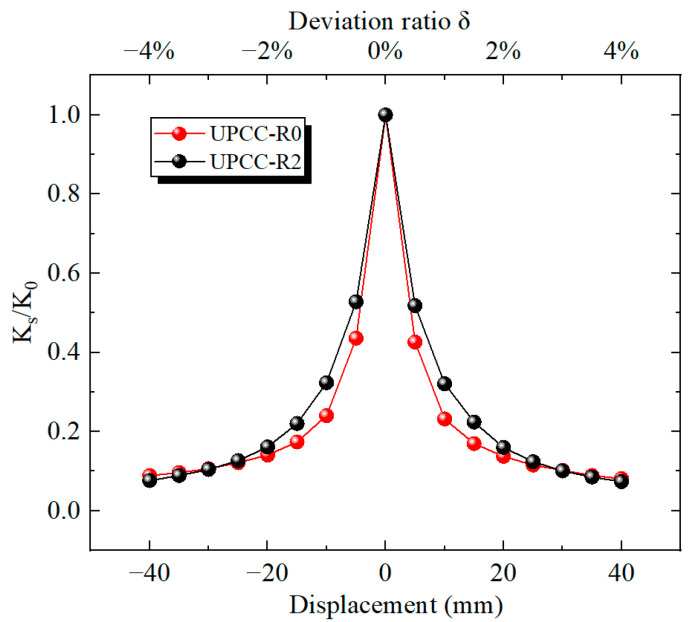
Stiffness degradation curves of two types of bridge piers.

Figure 15 shows the degradation stiffness curves of the piers without an external EREDL and an EREDL–PSCSFT. It can be seen from Figure 15 that with the increase in horizontal displacement from zero, the equivalent stiffness decreases during the whole loading process. Among them, the descending speed is the fastest at 0–15 mm, and the descending speed starts to slow down at 15–40 mm. At the initial stage of loading, the pier test piece with an EREDL set at the joint is obviously higher than the pier test piece without an external EREDL. However, with the increase in displacement load, the load displacement curve of the pier test piece without an external EREDL and the pier test piece with an EREDL set at the joint tend to coincide gradually, indicating that the setting of the EREDL can effectively reduce the stiffness degradation of the pier test piece; however, it has no impact on the final effective stiffness value of the pier.

### 4.6. Plastic Strain

**Figure 16:**
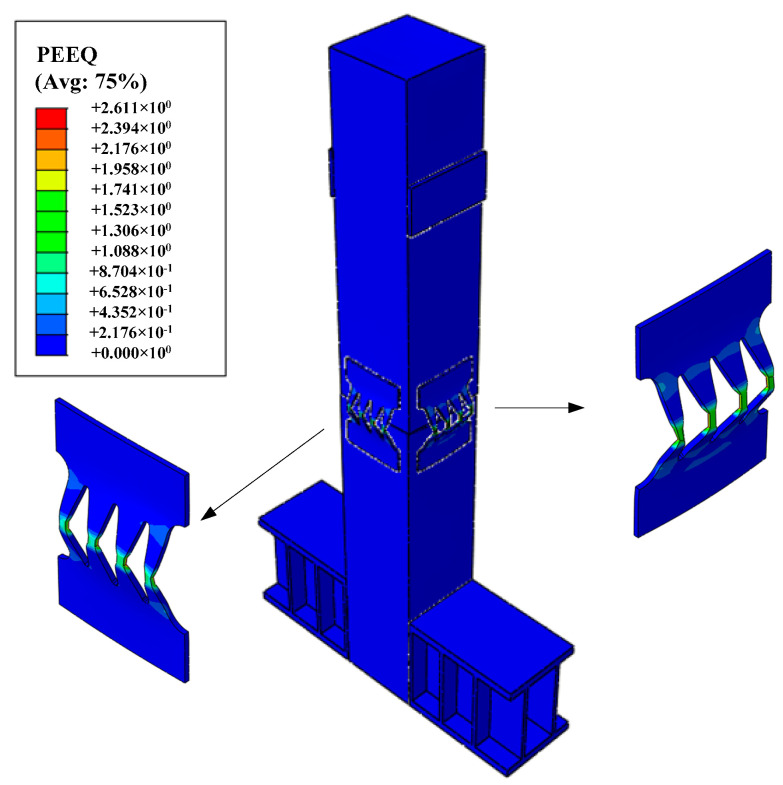
PEEQ (Equivalent plastic strain nephogram).

Figure 16 shows the equivalent plastic strain nephogram of the EREDL–PSCFST pier. It can be seen from Figure 16 that the plastic strain concentration of the EREDL-PSCFST pier occurs on the EREDL, which indicates that the EREDL can improve the energy consumption capacity of the PSCFST pier and can focus the damage on the EREDL. During an earthquake, the plastic deformation and dissipated energy of the pier mainly occur at the joints, and the EREDL around the steel tube section can be used for energy dissipation; however, the energy dissipation is mainly borne by the EREDL in the loading direction, so its plastic deformation is more obvious, and mainly concentrated in the middle part of the component. The connection between the two ends and the steel tube section is still in an elastic state, which is convenient for the replacement of the EREDL after an earthquake.

## 5. Seismic Performance and Influence Parameter Analysis

In the existing studies, the influence of the axial compression ratio on the seismic performance of PSCFST piers has been explored. This section mainly explores the impact of initial prestress, different EREDL strengths, and different thicknesses on the seismic performance of PSCFST piers.

### 5.1. Effect of Initial Prestress

In order to study the influence of initial prestress on the seismic performance of the EREDL–PSCFST pier, the pier models with initial prestress of 300 MPa, 600 MPa and 900 MPa are selected. The specific working conditions are shown in Table 7, in which the UPCC-R2 specimen model is the basic model.

**Figure 17:**
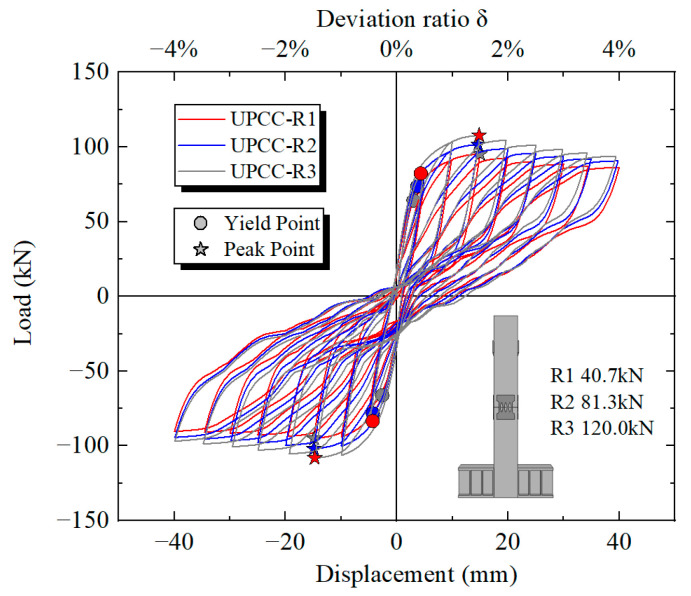
This is a figure. Schemes follow the same formatting.

**Figure 18:**
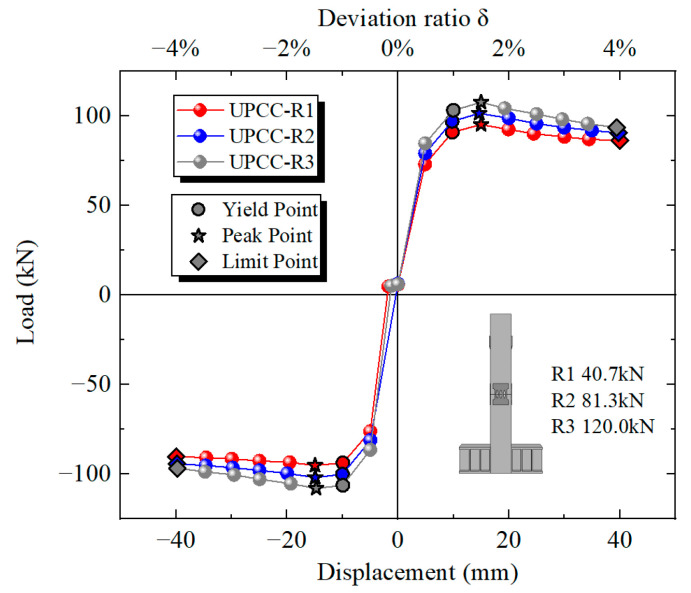
Skeleton curve under different initial prestress.

By comparing the hysteresis curve in Figure 17 and the skeleton curve in Figure 18, it can be found that with the increasing proportion of initial prestress of the piers, the maximum bearing capacity of piers also increases, but the increase amplitude is small. The maximum bearing capacity of a UPCC-R1 pier is 95.28 kN, and that of a UPCC-R2 pier is 101.56 kN, which is 6.28 kN higher than that of a UPCC-R1 pier; about 6.59% higher. The maximum bearing capacity of a UPCC-R3 pier is 107.73 kN, which is 13.07% higher than that of a UPCC-R1 pier, and 6.08% higher than that of a UPCC-R2 pier.

It can be seen from the Table 8 that with the increase in initial prestress, the ultimate displacement of the specimen decreases continuously, and the ductility decreases obviously, but the yield displacement of the three groups of specimens is basically the same. When the initial prestress increases from 40.7 kN to 120.0 kN, the ductility factor decreases by about 20.6%. Therefore, the initial prestress of the specimen should be slightly limited, such as limiting the minimum initial prestress.

**Figure 19:**
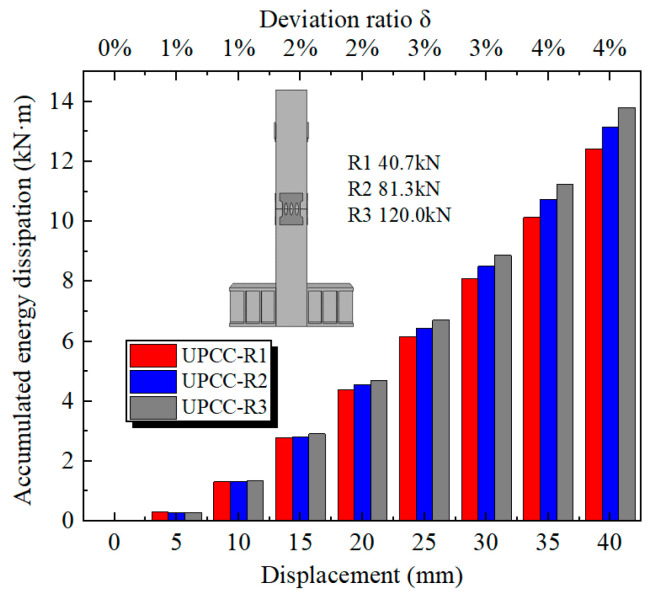
Cumulative energy dissipation curve under different initial prestress.

Comparing the cumulative energy consumption curves of piers under three different initial prestresses in Figure 19, it can be seen that the cumulative energy consumption of piers increases with the increase in initial prestress. The cumulative energy consumption of a UPCC-R1 pier is 12.42 kN · m, that of a UPCC-R2 pier is 13.16 kN · m, and that of a UPCC-R3 pier is 13.80 kN · m. Compared with a UPCC-R1 pier, the cumulative energy consumption of a UPCC-R2 pier has increased by 5.96%, and that of a UPCC-R3 pier has increased by 11.11%.

**Figure 20:**
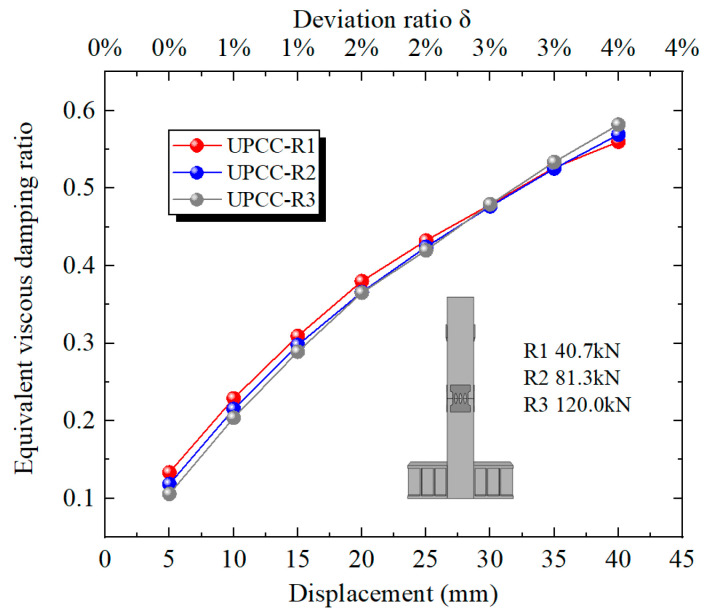
Equivalent viscous damping ratio under different initial prestress.

Figure 20 shows the equivalent viscous damping ratio of each stage of loading displacement in the loading process of different prestressed specimens. It can be seen from Figure 20 that, throughout the loading process, the equivalent viscous damping ratio curves of the three groups of specimens demonstrated an upward trend and gradually leveled off, and the equivalent viscous damping ratio of the three groups of specimens was close. When the offset rate is less than 3%, the equivalent viscous damping ratio of the R1 specimen is slightly higher, and the equivalent viscous damping ratio of R3 is slightly lower. However, when the offset rate is greater than 3%, the equivalent viscous damping ratio of the R3 specimen is slightly higher, and the equivalent viscous damping ratio of the R1 specimen is slightly lower. This shows that the initial prestress has little effect on the equivalent viscous damping ratio of the specimen, but the larger the initial prestress, the larger the equivalent viscous damping ratio at the initial stage of loading, and with the continuous increase in displacement loading, the rising speed is slower.

**Figure 21:**
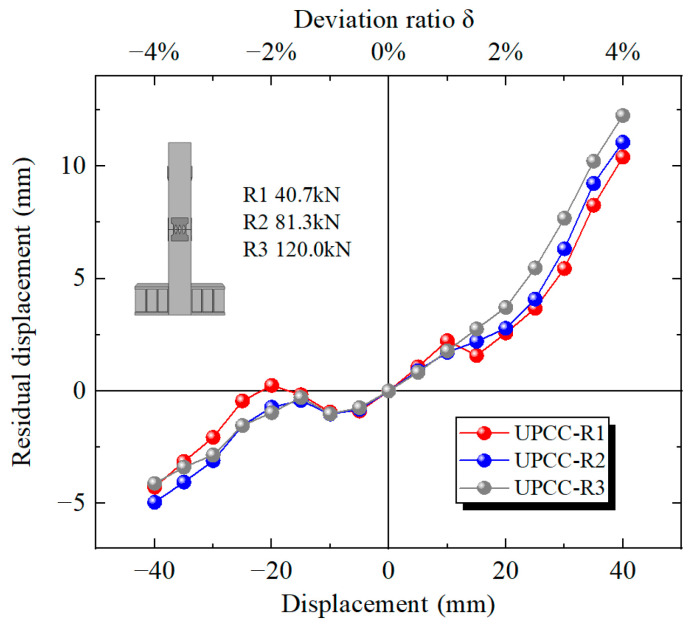
Residual displacement curves under different initial prestress.

It can be seen from the residual displacement loading displacement curves of three groups of pier specimens with different initial prestress in Figure 21 that when the displacement loading value is small, the residual displacement curves of the three groups of specimens basically coincide, indicating that the impact of initial prestress on the residual displacement of the pier can be ignored. When the displacement is loaded to 20 mm, the residual displacement of the specimen increases rapidly with the increase in displacement, and the greater the initial prestress, the greater the residual displacement of the specimen. When loading to 40 mm, the residual displacement of the specimen with initial prestress of 300 MPa is 10.404 mm, the residual displacement of the specimen with initial prestress of 600 MPa is 11.065 mm, and the residual displacement of the specimen with initial prestress of 900 MPa is 12.253 mm.

**Figure 22:**
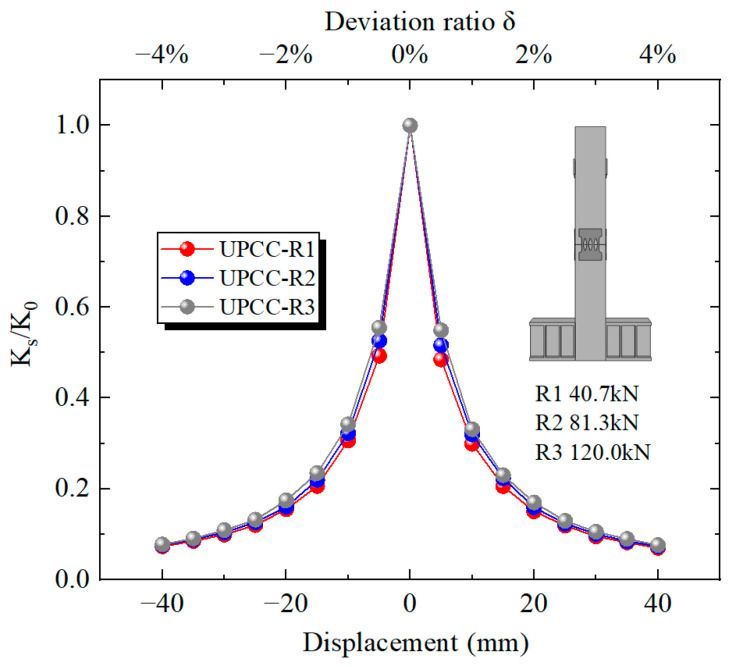
Stiffness degradation curve under different initial prestress.

It can be seen from Figure 22 that with the increase in horizontal displacement from zero, the equivalent stiffness decreases during the whole loading process. Among these, the descending speed is the fastest at 0–15 mm, and the descending speed starts to slow down at 15–40 mm. At the same time, in the whole loading stage, although the three groups of pier specimens with different initial prestress ratios are different, the displacement curves of the three groups of specimens basically coincide. This demonstrates that the initial prestress has little effect on the equivalent stiffness of the pier.

### 5.2. Effect of Steel Yield Strength of EREDL

In order to study the influence of EREDL strength on the seismic performance of piers, pier models with EREDL strengths of Q120, Q235, Q345 and Q425 are selected for research and analysis. The design parameters are shown in Table 9.

**Figure 23:**
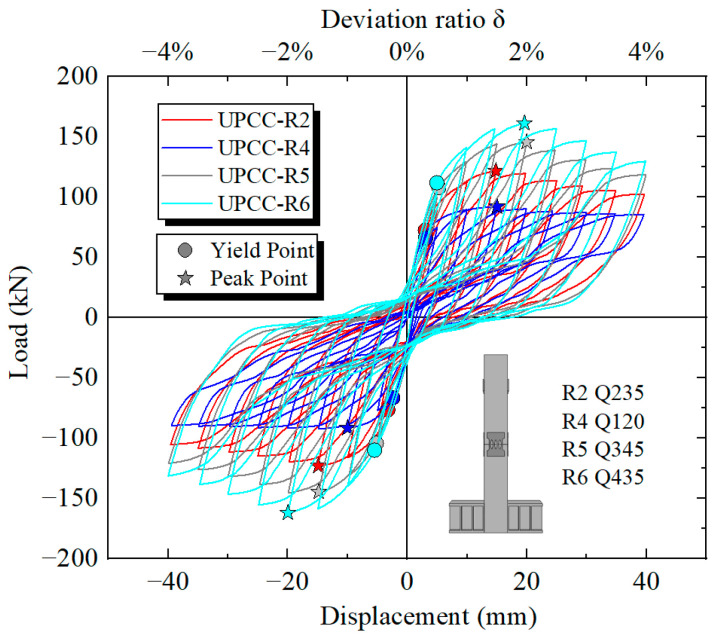
Hysteretic curves under different steel yield strength of EREDL.

**Figure 24:**
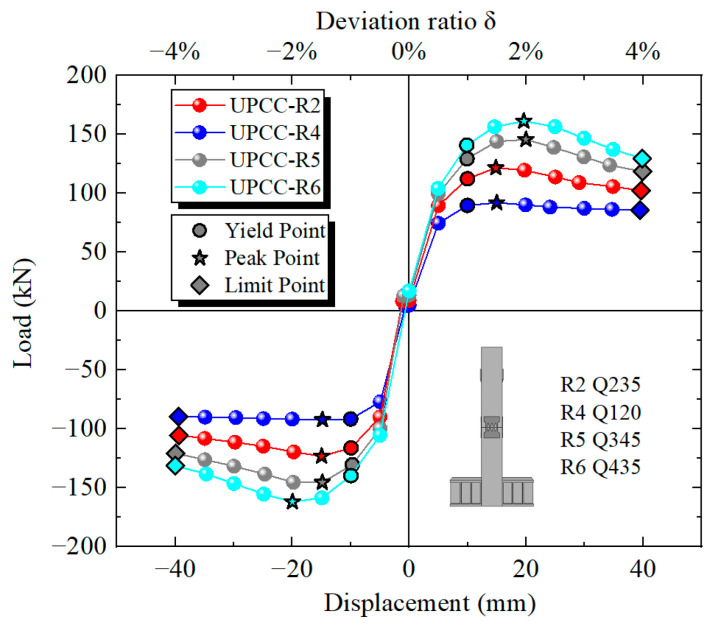
Skeleton curves under different steel yield strength of EREDL.

By comparing the hysteresis curve in Figure 23 and the skeleton curve in Figure 24, it can be found that the maximum bearing capacity of the pier is also increasing with the increasing EREDL strength. When the EREDL strength is Q195, the maximum bearing capacity of the pier is 92.1 kN. when the EREDL strength is Q235, the maximum bearing capacity of the pier is 121.7 kN, which is 29.6 kN higher than the R4 bearing capacity; about 32.1%. when the EREDL strength is Q345, the maximum bearing capacity of the pier is 145.7 kN, which is 53.6 kN higher than the R4 maximum bearing capacity; about 58.2%. The bearing capacity of R2 is increased by 24 kN; about 19.7%. When the EREDL strength is Q435, the maximum bearing capacity of the pier is about 161.3 kN, which is 69.2 N, about 75.1% higher than the maximum bearing capacity of specimen R4, 39.6 kN, about 31.2% higher than the maximum bearing capacity of specimen R2, and 15.6 kN, about 10.7% higher than the maximum bearing capacity of specimen R5.

In addition, when Q195 and Q235 reach the maximum bearing capacity, the load displacement is 15 mm, and when Q345 and Q435 reach the maximum bearing capacity, the load displacement is 20 mm. It can be seen that when the EREDL strength reaches a certain extent, the displacement load required for the specimen to reach the maximum bearing capacity also increases to a certain extent.

It can be seen from Table 10 that with the increase in the strength of the connector, the ultimate displacement of the specimen decreases continuously, and the ductility decreases obviously at the beginning. However, when the strength of the connector increases to a certain extent, the ductility of the specimen is almost no longer affected by the strength of the connector and remains at about 5.96. The yield displacement of the three groups of specimens is basically the same, and the strength of the connector has no effect on the yield displacement of the specimen.

**Figure 25:**
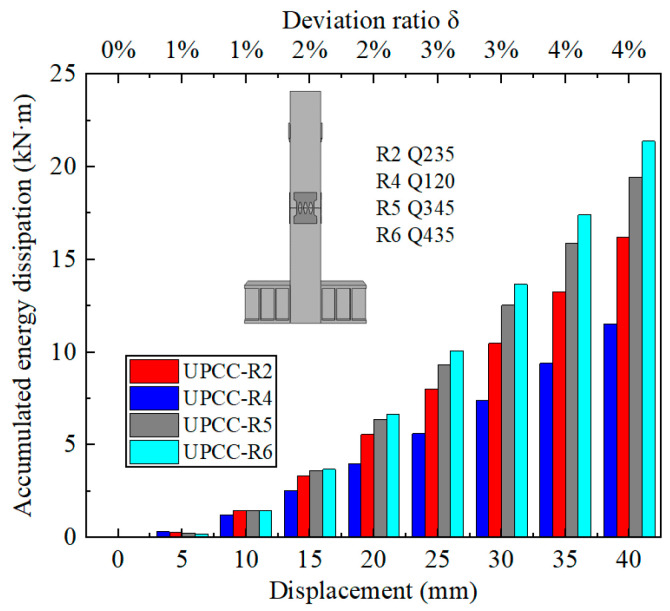
Cumulative energy dissipation curves under different steel yield strength of EREDL.

It can be seen from Figure 25 that with the increase in EREDL strength, the cumulative energy consumption of piers also increases. When the load reaches 40 mm, the final cumulative energy consumption of each group of test pieces, the strength increase in each test piece compared with R4 test piece, and the cumulative energy consumption increase is shown in Table 11.

The following two points can be seen in Figure 25 and Table 11: (1) There is a certain gap in the cumulative energy consumption of the test pieces under different EREDL strengths, and the gap between the cumulative energy consumption of each test piece continues to widen with the increasing displacement load. (2) With the increase in EREDL strength, although the cumulative energy consumption of the specimen is also increasing, the increasing range is gradually decreasing.

**Figure 26:**
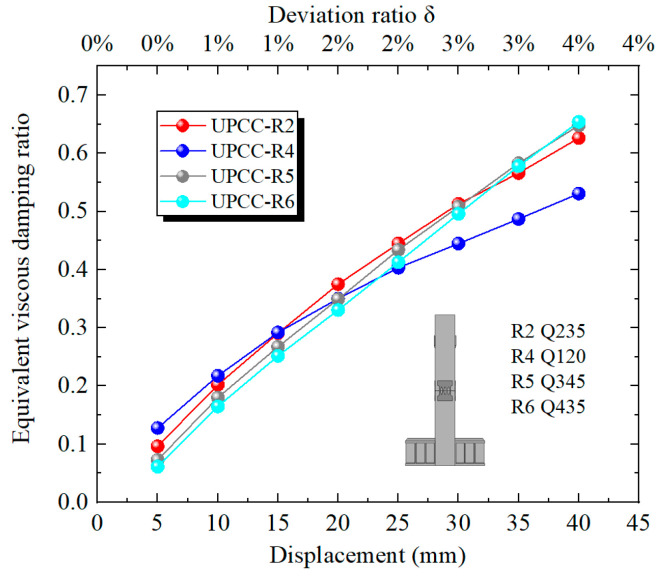
Equivalent viscous damping ratio under different steel yield strength of EREDL.

Figure 26 shows the equivalent viscous damping ratio of each stage of loading displacement during the loading process of different strength connector specimen models. It can be seen from Figure 26 that the equivalent viscous damping ratio curves of the four groups of specimens show an upward trend during the loading process. When the offset rate is less than 1%, the equivalent viscous damping ratio of the R4 specimen is slightly higher, and the equivalent viscous damping ratio of the R6 is slightly lower. However, when the offset rate is greater than 3%, the equivalent viscous damping of R2, R5 and R6 is relatively close. However, the equivalent viscous damping ratio of the R6 specimen is slightly higher than that of the R2 and R5 specimens, and the equivalent viscous damping ratio of the R4 specimen is obviously lower. This shows that when the strength of the connector is in a lower range, its size has a greater impact on the equivalent viscous damping ratio of the specimen. However, when the strength of the connector is greater than this range, the influence of its size on the equivalent viscous damping ratio of the specimen is weak, and the equivalent viscous damping of the specimens with different strength connectors is relatively close.

**Figure 27:**
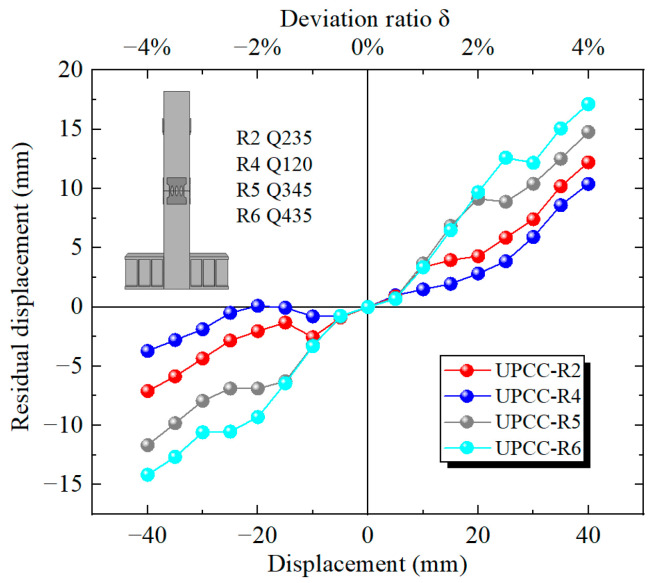
Residual displacement under different steel yield strength of EREDL.

According to the residual displacement loading displacement curves of three groups of different initial prestressed pier specimens in Figure 27, when the initial reciprocating loading displacement reaches 5 mm, the residual displacement of four groups of specimens is basically the same, indicating that under small displacement loading, the influence of EREDL strength on the residual displacement of specimens can be ignored. When the displacement is loaded to 10 mm, the residual displacement of R2, R5 and R6 groups of specimens with higher EREDL strength is basically the same, while the residual displacement of R4 is significantly smaller than that of the other three groups of specimens. When the load reaches 15 mm, the residual displacement of R5 and R6 is approximately the same, while that of R2 is smaller than that of R5 and R6. When the load reaches 25 mm, the residual displacement of the R5 and R6 groups of specimens starts to differ, and the residual displacement of specimens with smaller EREDL strength is smaller. From the above analysis, it can be seen that in the initial stage of loading, the strength of EREDL has no obvious effect on the residual displacement of the test piece. However, with the gradual increase in displacement load, the residual displacement of the test pieces with different EREDL strengths starts to differ. The smaller the strength of EREDL, the smaller the load displacement whose residual displacement growth amplitude starts to decrease.

**Figure 28:**
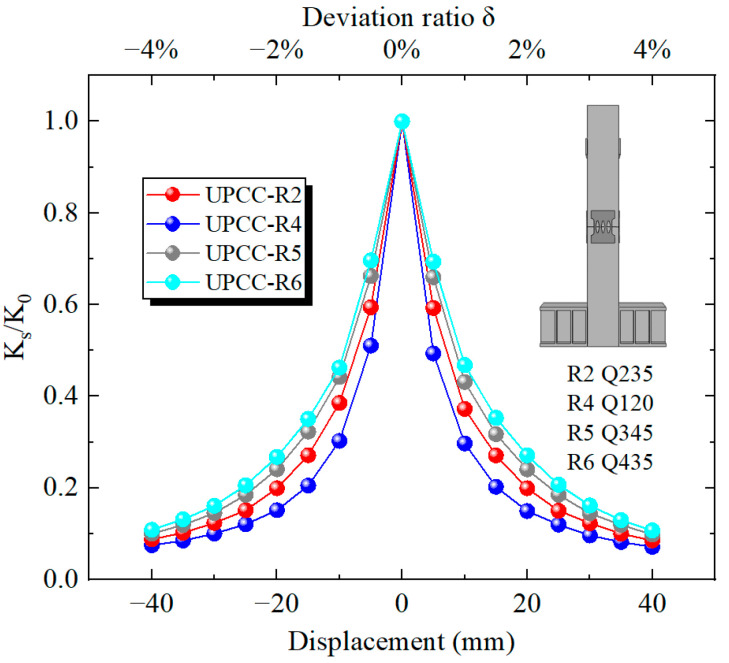
Degradation stiffness under different steel yield strength of EREDL.

It can be seen from Figure 28 that with the increase in horizontal displacement from zero, the equivalent stiffness decreases during the whole loading process. Among them, the descending speed is the fastest at 0–15 mm, and the descending speed starts to slow down at 15–40 mm. At the same time, in the whole loading stage, the four groups of different initial prestressed pier specimens are different. The equivalent stiffness of specimen R6, i.e., the specimen with EREDL strength of Q435, is always at a large level, while the equivalent stiffness of specimen R4, i.e., the specimen with EREDL strength of Q195, is always at a small level. It can be seen that the strength of the EREDL has a significant impact on the equivalent stiffness of the test piece. The greater the strength of the EREDL, the greater the equivalent stiffness of the pier test piece in the earthquake process.

### 5.3. Effect of EREDL Thickness

EREDL is arranged at the joints between segments, and the seismic performance of each pier is different according to the thickness of the EREDL. In order to study the influence of EREDL thickness on the seismic performance of piers, pier models with EREDL thicknesses of 5 mm, 8 mm and 10 mm are selected for research and analysis. The design parameters are shown in Table 12.

**Figure 29:**
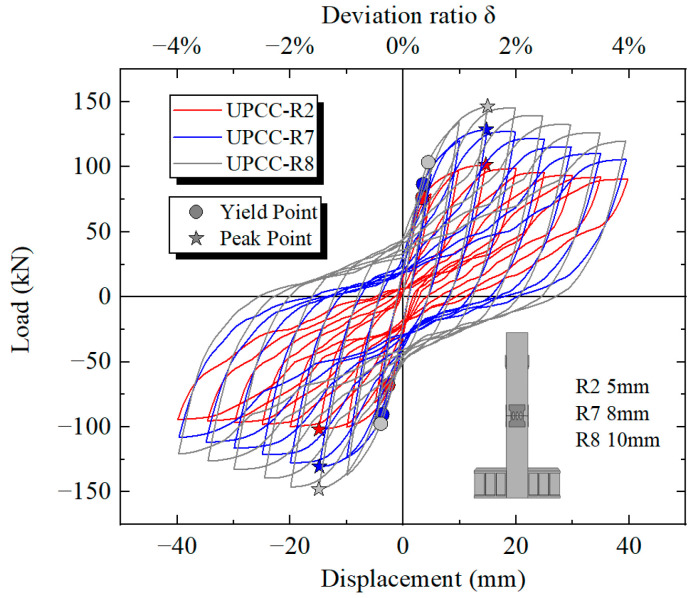
Hysteretic curves under different thicknesses of EREDL.

By comparing the cumulative energy consumption curves of piers with three different thicknesses of diamond-shaped perforated EREDL, it can be seen that with the increase in EREDL thickness, the cumulative energy consumption capacity of piers also increases. The accumulated energy consumption of the R2 specimen is 13.16 kN · m, the R7 specimen is 23.40 kN · m, and the R8 specimen is 31.13 kN · m. Compared with the R2 pier specimen, the accumulated energy consumption of the R7 specimen is increased by 77.81%, and that of the R8 specimen is increased by 136.55%. This conclusion can also be seen from the hysteretic curve in Figure 29: with the increase in the thickness of the diamond shaped opening EREDL, the overall stiffness of the pier increases, and the hysteretic curve becomes fuller. Therefore, the energy dissipation capacity of the pier is improved.

**Figure 30:**
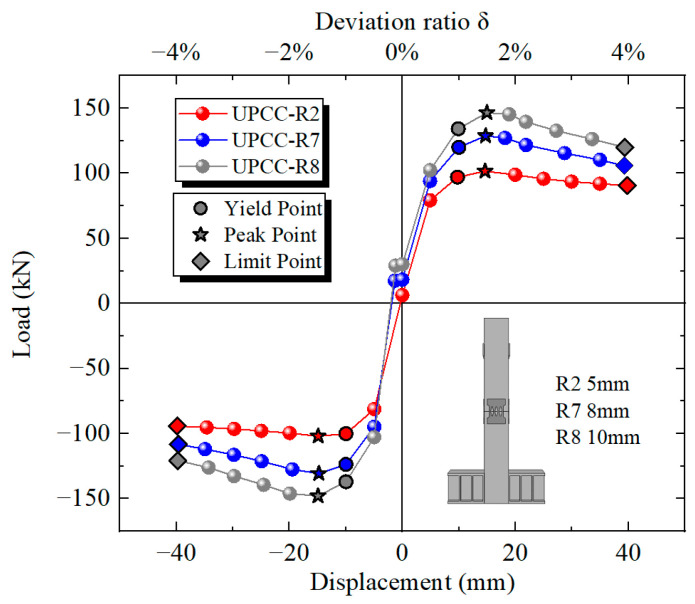
The skeleton curves under different thickness of EREDL.

By comparing the hysteresis curve in Figure 29 and the skeleton curve in Figure 30, it can be found that the maximum bearing capacity of the pier is also increasing with the increasing thickness of EREDL. The maximum bearing capacity of the R2 specimen is 101.56 kN, that of the R7 specimen is 128.77 kN (which is about 26.79% higher than that of R2 specimen), and that of R8 specimen is 146.58 kN (which is about 44.33% higher than that of R2 specimen). In addition, with the continuous increase in EREDL thickness, the hysteretic curve of the pier is fuller and the arched characteristics are more obvious, indicating that the energy dissipation capacity of the pier is significantly enhanced.

It can be seen from Table 13 that the ultimate displacement and ductility coefficient of specimens R7 and R8 are the same, while the ultimate displacement and ductility coefficient of specimen R2 is significantly higher than those of specimens R7 and R8. When the thickness of connectors increases to a certain extent, the ductility of specimens is no longer affected by the thickness of connectors. The yield displacement of the three groups of specimens is basically the same, and the thickness of the connector has no effect on the yield displacement of the specimen.

**Figure 31:**
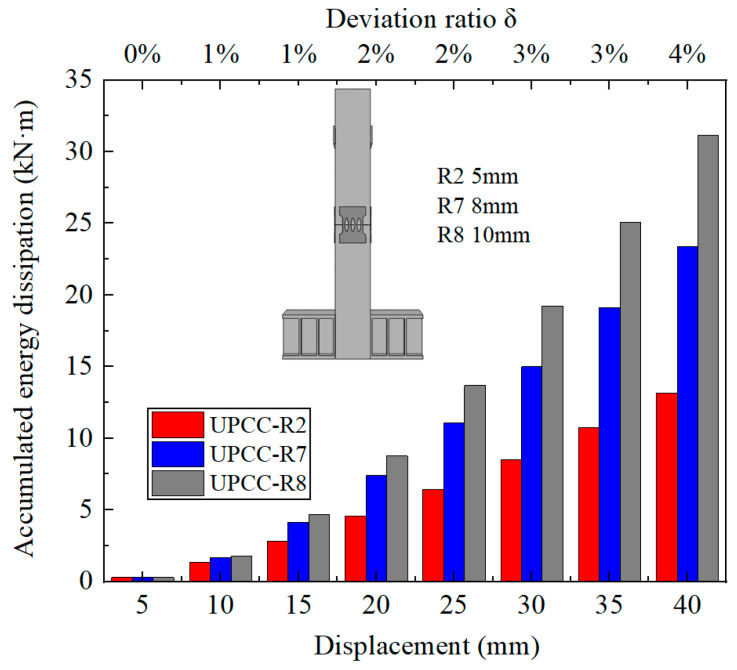
Cumulative energy dissipation curve under different EREDL thickness.

By comparing the cumulative energy consumption curves of piers with three different thicknesses of diamond shaped perforated EREDL in Figure 31, it can be seen that with the increase of EREDL thickness, the cumulative energy consumption capacity of piers also increases. The accumulated energy consumption of R2 specimen is 13.16kN · m, R7 specimen is 23.40kN · m, and R8 specimen is 31.13kN · m. Compared with R2 pier specimen, the ac-cumulated energy consumption of R7 specimen is increased by 77.81%, and that of R8 specimen is increased by 136.55%. This conclusion can also be seen from the hysteretic curve in Figure 29: with the increase of the thickness of the diamond shaped opening EREDL, the overall stiffness of the pier increases, and the hysteretic curve becomes fuller. Therefore, the energy dissipation capacity of the pier is improved.

**Figure 32:**
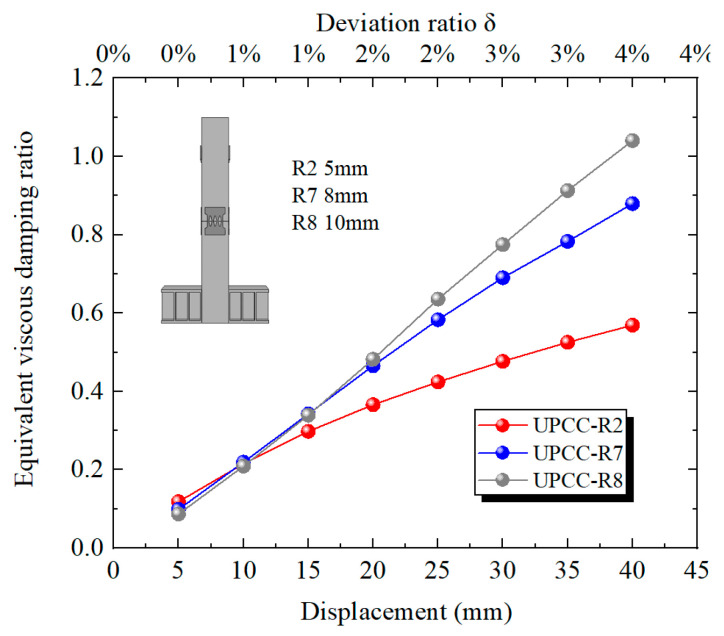
Equivalent viscous damping ratio under different thicknesses of EREDL.

Figure 32 shows the equivalent viscous damping ratio of each stage loading displacement of the specimen model with different connector thicknesses during the loading process. It can be seen from Figure 32 that, during the loading process, the equivalent viscous damping ratio curves of the three groups of specimens show an upward trend, however, the equivalent viscous damping ratio of the R8 specimen rises faster, and the equivalent viscous damping ratio of the R2 specimen rises slower. When the offset rate is less than 1%, the equivalent viscous damping ratio of the R2 specimen is slightly higher, and the equivalent viscous damping ratio of the R8 is slightly lower. However, when the offset rate is greater than 1%, the equivalent viscous damping ratio of the R8 specimen is significantly higher than that of the other two groups, and the equivalent viscous damping ratio of the R2 specimen is the lowest. This demonstrates that when the offset rate is small, the thickness of the connector has little effect on the equivalent viscous damping ratio of the specimen. However, with the increase in displacement loading, the opening at the joint increases continuously, and the connector is stretched. The influence of the thickness of the connector on the equivalent viscous damping ratio of the specimen gradually increases. The greater the thickness of the connector, the greater the equivalent viscous damping ratio of the specimen.

**Figure 33:**
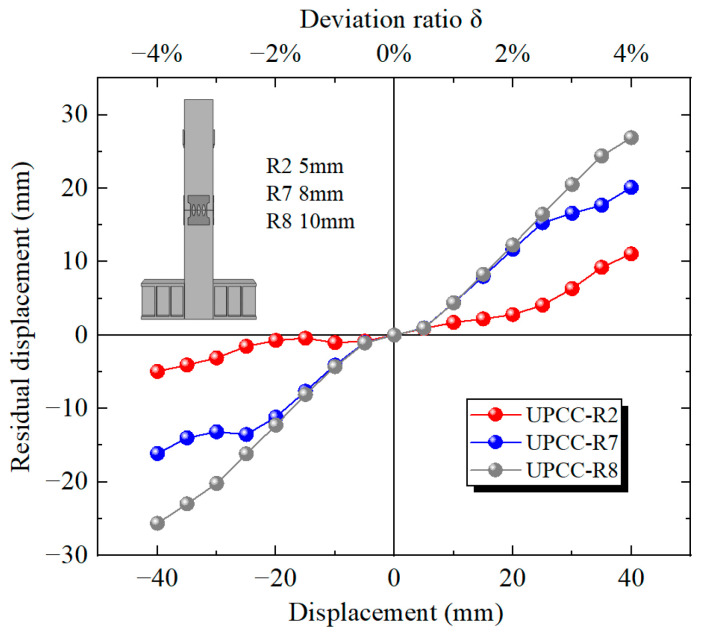
Residual displacement curves under different thicknesses of EREDL.

It can be seen from the residual displacement curve in Figure 33 that as the thickness of EREDL increases, the residual displacement of the pier also increases significantly. The residual displacement of the R2 test piece is 11.06 mm, that of the R7 test piece is 20.12 mm, and that of the R8 test piece is 26.88 mm. Compared with the R2 specimen, the residual displacement of the R7 specimen increased by 81.92%, and that of the R8 specimen increased by 143.04%.

When the displacement reaches 5 mm, the residual displacement of the three EREDL piers with different thicknesses is almost the same. This is because when the displacement is loaded to 5 mm, the pier as a whole is in the elastic stage, and there is almost no plastic deformation, therefore the residual displacement of the three is basically close to zero. This can also be seen from the hysteresis curve and skeleton curve. However, when the displacement is slowly loaded to 10 mm, the residual displacement of the R2 specimen starts to decrease significantly compared with the other two groups of piers. Before loading to 20 mm, the residual displacement of the R7 specimen and the R8 specimen is basically the same, and they coincide as a straight line. The residual displacement of the R7 specimen began to decrease compared with that of the R8 specimen until it was loaded to 25 mm. It shows that the R2 test piece can better restrain the generation of residual displacement of the bridge pier and play a role in restraining the generation of residual displacement of the bridge pier earlier.

**Figure 34:**
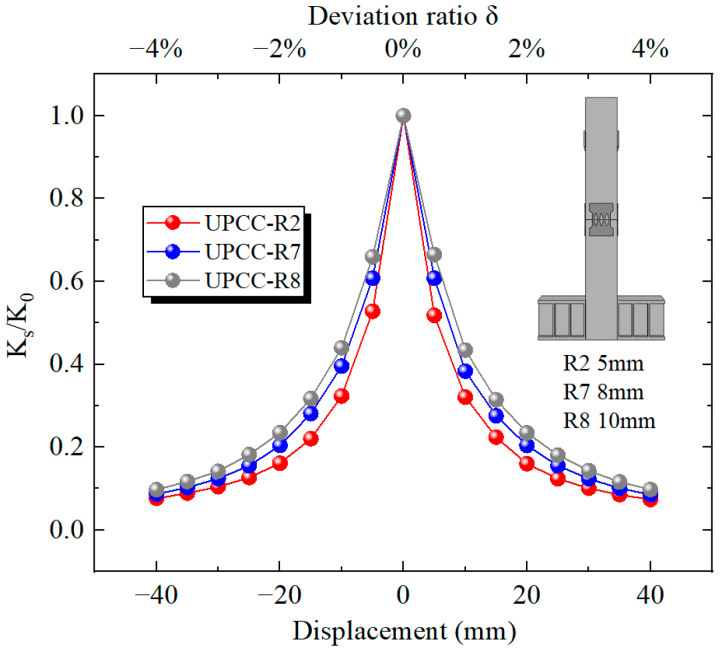
Degradation stiffness under different thicknesses of EREDL.

It can be seen from Figure 34 that the equivalent stiffness decreases during the whole loading process as the horizontal displacement increases from zero. Among them, the descending speed is the fastest at 0–10 mm, and the descending speed starts to slow down at 10–40 mm. At the same time, during the whole loading stage, the degradation stiffness of the R8 specimen is always at a relatively large level, followed by the R7 specimen, and the R2 specimen is always at a relatively small level. The above results show that the increase in EREDL thickness can effectively increase the equivalent stiffness of the pier.

## 6. Analysis of Bending Bearing Capacity of Test Piece

The shear-bearing capacity of precast segmental piers is mainly provided by the mutual friction between the concrete at the joints. The Formula [29] for calculating the shear resistance at the pier joints of precast concrete segments is as follows:

(21) $$V_{j}=A_{j}\mu \sigma_{n}$$

where:

$V_{j}$—shear resistance of joint surface
$A_{j}$—Contact area of joint surface
μ—Friction coefficient
$\sigma_{n}$—Normal stress value at joint surface.

The shear bearing capacity of piers proposed in this paper is provided not only by the mutual friction between the concrete at the joints, but also by external energy dissipators. Therefore, the above formula is supplemented as follows:

(22) $$V_{j}=A_{j}\mu \sigma_{n}+nA_{k}f_{v}$$

where:

$A_{k}$—sectional area of the energy dissipator
$f_{v}$—Shear strength of energy dissipator steel
n—Number of energy consumers
$\sigma_{n}$—Provided by axial pressure and prestress
μ—according to AASHTO American Specification [30], μ is 0.6.

Substituting the low-cycle reciprocating test data of a CFST pier into Formula (22), 451.21 kN is calculated, which is much greater than the ultimate load of 161.3 kN found in the test. This shows that no shear failure occurred to the pier specimen in the low cycle reciprocating test, so the CFST pier is still.

**Figure 35:**
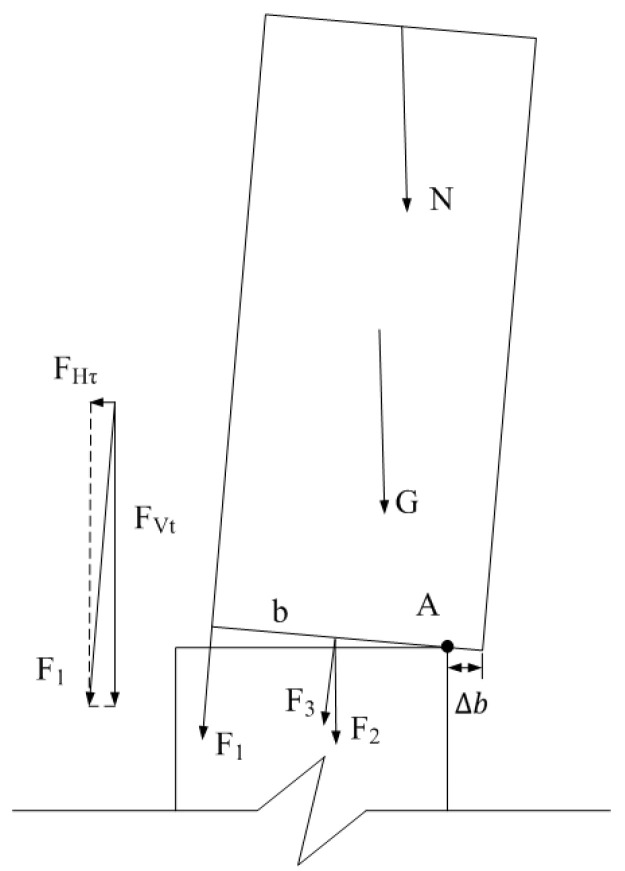
Ultimate stress state of pier bending.

In the bending deformation state, stress analysis is carried out for its bending limit state (as shown in Figure 35, moment is calculated for point A), and the bending bearing capacity reference Formula (21) for CFST piers with external dampers is obtained.

(23) $$M_{u}=F_{1}\left(b-\Delta b\right)+\left(G+N+F_{2}+F_{3}\right)\left(\frac{b}{2}-\Delta b\right)$$

where:

$F_{1}$,$F_{2}$—force exerted by energy dissipators at different positions on pier segments
$F_{H\tau }$—The shear stress of $F_{1}$ in the horizontal direction
$F_{Vt}$—The tensile stress of $F_{1}$ in the vertical direction
$F_{3}$—Tension generated by initial prestress and prestressed reinforcement when the pier segment rotates
G—Dead weight of concrete-filled steel tube segments, calculated as 20 kN
N—Axial force
b—Section width of concrete-filled steel tube sections
Δb—the relative slip distance between the two segments. According to the finite element simulation results, ∆*b* is within the loading displacement range of about 6%~12% bending limit state, and the loading displacement value of 9% bending limit state is 0.45 mm.

Formula (24) is derived from Formula (23) in one step:

$$F_{1}=2F_{2}=\varepsilon_{y}E_{s1}\lambda A$$

$$F_{3}=\left(p+\frac{\varepsilon_{y}E_{s2}A_{s}}{2}\right)$$

$$N=aN_{0}$$

(24) $$M_{u}=\varepsilon_{y}E_{s1}\lambda A\left(b-\Delta b\right)+\left(G+aN_{0}+\frac{\varepsilon_{y}E_{s1}\lambda A}{2}+\left(p+\frac{\varepsilon_{y}E_{s2}A_{s}}{2}\right)\right)\left(\frac{b}{2}-\Delta b\right)$$

where:

λ—section ratio
A—sectional area of concrete-filled steel tube segments; 4 in this paper × 104 mm^2^;
a—Axial compression ratio
$N_{0}$—Ultimate bearing capacity of concrete-filled steel tube pier, $N_{0}=f_{c}A_{c}+f_{y}^{\prime }A_{s}=2405.8\;kN$p—Initial prestress
p—Initial prestress
$\varepsilon_{y}$—Yield strain of energy dissipator steel
$E_{s1}$—EREDL elastic modulus; 1.86 GPa is taken in this paper
$E_{s2}$—Elastic modulus of prestressed reinforcement, 1.95 GPa
$A_{s}$—sectional area of prestressed reinforcement, 139 mm^2^.

Substituting all finite element parameters into Formula (24), it can be found that the average value of the ratio between the simulated value of the formula and the calculated value of the theoretical formula of the eight finite element models is 1.009. The specific finite element model parameters and the comparison between the simulated and calculated values are shown in Table 14. Although there is some error between the finite element simulation value and the theoretical formula calculation value, it is basically kept within 5%. It can be seen from the comparison between the two that the initial prestress has little impact on the bearing capacity of the bridge pier. Although the initial prestress is increasing, the ratio between the two is basically stable at 1.043. The strength and thickness of EREDL has a significant impact on the bearing capacity of the bridge pier. With the increase in the thickness and strength of EREDL, the deviation gradually increases slowly.

## 7. Conclusions

This paper first verified the effectiveness of finite element simulation through tests, and then found that the external energy dissipators can greatly improve the energy dissipation capacity, lateral bearing capacity, and other seismic performance of PSCFST piers through finite element analysis, and the following conclusions were obtained:

(1) When the initial prestress is 600 MPa, the EREDL strength is Q235, and the thickness is 5 mm, the ductility coefficient and residual displacement increase with the increase in initial prestress. When the initial prestress increases from 40.7 kN to 120.0 kN, the ductility factor decreases by about 20.6%. However, the equivalent viscous damping of specimens with different initial prestress is close;
(2) Under the low cycle reciprocating horizontal displacement loading, EREDL–PSCFST piers mainly dissipate the energy with the plastic deformation of EREDL, and the pier damage is mainly concentrated in EREDL during the energy dissipation. EREDL can effectively reduce the damage of the pier steel tube concrete segment. Furthermore, the easy-to-replace EREDL makes the rapid repair of piers after the earthquake possible, and enhances the recoverable function of piers;
(3) In this study, with the increase in EREDL strength in the range of Q120–Q425, the seismic performance of piers has been significantly improved. The bearing and energy capacity of specimens tends to increasing with the EREDL strength increases, however, the residual displacement also increases. Interestingly, with the increasing strength of EREDL, the effect gradually weakened;
(4) The theoretical formula for calculating the flexural capacity of EREDL–PSCFST piers proposed in this paper takes into account the relative slip between segments during loading. The average ratio of the calculated value to the finite element simulation value is 1.009, and the error is basically maintained within 5%. The calculated value is more consistent with the simulated value, which can provide a reference for the calculation of the flexural capacity of EREDL–PSCFST piers in practical engineering.

The piers in this paper have a self-recovery function which can effectively reduce the difficulty and cost of pier maintenance after an earthquake. The piers thus have good social and economic benefits. Consequently, it is also necessary to conduct quantitative analyses on PSCFST piers with external energy dissipators in order to obtain the optimal number of related energy dissipators.

## Figures and Tables

**Table 1:** The specific working condition of each bridge pier finite element model.

Specimen	EREDL	Initial Prestress (MPa)	Ratio of Axial Compression Stress to Strength	Steel Type of EREDL	Thickness of EREDL (mm)
UPCC-R0	NO	600	0.15	-	-
UPCC-R1	YES	300	0.15	Q235	5
UPCC-R2	YES	600	0.15	Q235	5
UPCC-R3	YES	900	0.15	Q235	5
UPCC-R4	YES	600	0.15	Q120	5
UPCC-R5	YES	600	0.15	Q345	5
UPCC-R6	YES	600	0.15	Q425	5
UPCC-R7	YES	600	0.15	Q235	8
UPCC-R8	YES	600	0.15	Q235	10

**Table 2:** Parameters of ABAQUS concrete plastic model.

ψ	ϵ	$f_{b0}/f_{c0}$	$K_{c}$	μ
30	0.1	1.16	0.6667	0.0005

**Table 3:** Axial tensile strength and axial compressive strength of concrete.

Test Block	Compressive Strength (MPa)	Tensile Strength (MPa)
1	42.8	2.35
2	43.1	2.43
3	41.6	2.40
Average	42.5	2.39

**Table 4:** Comparison between simulated values and experimental values.

Comparative Item	Horizontal Bearing Capacity/kN Side Shift 6.2%	Residual Displacement/mm Side Shift 6.2%	Equivalent Stiffness/(kN·mm^−1^) Side Shift 6.2%	Energy Consumption/(kN·mm) Side Shift 6.2%
Experimental values	74.1	5.8	1.2	12.0
Finite element values	62.6	8.2	1.0	16.2
Rate	0.8	1.4	0.8	1.2

**Table 5:** Performance turning point of skeleton curve.

Specimen	UPCC-R0	UPCC-R2
Yield strength (kN)	50.32	97.04
Yield displacement (mm)	24.50	9.76
Peak load capacity (kN)	57.40	101.56
Peak displacement (mm)	39.70	14.65 mm
Ductility factor	1.62	1.50

**Table 6:** Energy dissipation performance index.

Specimen	E	$h_{e}$
UPCC-R0	0.116	0.018
UPCC-R2	0.728	0.116

**Table 7:** Bridge pier working conditions under different initial prestress.

Specimen	Initial Prestress (MPa)	Steel Type of EREDL	Thickness of EREDL (mm)
UPCC-R1	300	Q235	5
UPCC-R2	600	Q235	5
UPCC-R3	900	Q235	5

**Table 8:** Ductility coefficient of specimens under different initial prestress.

Specimen	Peak Carrying Capacity	Yield Displacement	Displacement (90% Peak Carrying Capacity)/mm	Ductility Factor Δu
UPCC-R1	95.27	4.87	40	8.21
UPCC-R2	101.56	4.9	37.04	7.56
UPCC-R3	107.73	4.91	32.02	6.52

**Table 9:** Bridge pier working conditions under different steel yield strength of EREDL.

Specimen	Initial Prestress (MPa)	Steel Type of EREDL	Thickness of EREDL (mm)
UPCC-R2	600	Q235	5
UPCC-R4	600	Q120	5
UPCC-R5	600	Q345	5
UPCC-R6	600	Q425	5

**Table 10:** The ductility coefficient of specimens under EREDL with different yield strength.

Specimen	Peak Carrying Capacity	Yield Displacement	Displacement (90% Peak Carrying Capacity)/mm	Ductility Factor Δu
UPCC-R2	101.56	4.90	37.04	7.56
UPCC-R4	92.075	4.98	40	8.03
UPCC-R5	145.67	4.98	29.78	5.98
UPCC-R6	161.25	5.02	29.92	5.96

**Table 11:** Final cumulative energy consumption of each specimen and the increase range of specimen strength and cumulative energy.

Specimen	Cumulative Energy Dissipation (kN·m)	The Increase Range of Strength	The Increase Range of Cumulative Energy
UPCC-R4	11.5	-	-
UPCC-R2	16.2	20.5%	40.4%
UPCC-R5	19.4	76.9%	68.5%
UPCC-R6	21.4	123%	85.4%

**Table 12:** Bridge pier working conditions under different thickness of EREDL.

Specimen	Initial Prestress (MPa)	Steel Type of EREDL	Thickness of EREDL (mm)
UPCC-R2	600	Q235	5
UPCC-R7	600	Q235	8
UPCC-R8	600	Q235	10

**Table 13:** The ductility coefficient of specimens under EREDL with different thickness.

Specimen	Peak Carrying Capacity	Yield Displacement	Displacement (90% Peak Carrying capacity)/mm	Ductility Factor Δu
UPCC-R2	101.56	4.90	37.04	7.56
UPCC-R7	128.79	4.93	28.17	5.71
UPCC-R8	146.58	4.90	27.97	5.71

**Table 14:** Comparison between simulated values and calculated values of flexural bearing capacity.

Model Number	Section Ratio	Axial Compression Ratio	Initial Prestress (MPa)	$\mathrm{Finite}\;\mathrm{Element}\;\mathrm{Value}\;M_{u,\vartheta }\;(\mathrm{kN}\cdot m)$	$\mathrm{Calculated}\;\mathrm{Value}\;M_{u,c}\;(\mathrm{kN}\cdot m)$	$M_{u,\vartheta }/M_{u,c}$
UPCC-R1	2%	0.15	300	47.6	45.64	1.043
UPCC-R2	2%	0.15	600	50.8	49.26	1.031
UPCC-R3	2%	0.15	900	53.9	52.23	1.032
UPCC-R4	2%	0.15	600	46.0	43.81	1.050
UPCC-R5	2%	0.15	600	72.0	69.94	1.029
UPCC-R6	2%	0.15	600	78.2	81.47	0.953
UPCC-R7	3%	0.15	600	64.4	67.58	0.953
UPCC-R8	4%	0.15	600	73.3	74.78	0.980

## Data Availability

All data, models, or code supporting the results of this study are available from the corresponding authors upon reasonable request.
